# Marine-Derived Polysaccharides Against Ulcerative Colitis: Sources, Structures, and Multi-Target Mechanisms

**DOI:** 10.3390/md24100332

**Published:** 2026-09-23

**Authors:** Dandan Zheng, Xuefei Wang, Yudi Xiang, Shuoshi Wei, Ruihua Chen, Qiang Lu, Cailan Li

**Affiliations:** 1 Department of Pharmacology, Zunyi Medical University, Zhuhai Campus, Zhuhai 519041, China; 2 Department of Pharmaceutical Sciences, Zunyi Medical University, Zhuhai Campus, Zhuhai 519041, China; 3 Key Laboratory of Basic Pharmacology of Ministry of Education and Joint International Research Laboratory of Ethnomedicine of Ministry of Education, Zunyi Medical University, Zunyi 563000, China; 4 Key Laboratory of Basic Pharmacology of Guizhou Province and School of Pharmacy, Zunyi Medical University, Zunyi 563000, China

**Keywords:** marine polysaccharides, ulcerative colitis, molecular mechanism, structural features, extraction

## Abstract

Marine-derived polysaccharides have emerged as promising bioactive candidates for alleviating ulcerative colitis (UC), a chronic inflammatory bowel disease with limited therapeutic options. This review systematically summarizes recent advances in the sources, preparation methods, structural features, and multi-target mechanisms of these polysaccharides from seaweeds, marine animals, and microorganisms. Key physicochemical parameters, including molecular weight, monosaccharide composition, glycosidic linkages, and sulfation patterns, critically influence their anti-colitis efficacy. Mechanistically, these macromolecules synergistically restore gut microbiota homeostasis (e.g., promoting short-chain fatty acids and secondary bile acids), suppress inflammatory pathways (NF-κB, MAPK, and NLRP3), regulate immune responses (inhibiting dendritic cell and Th1/Th17 activation), alleviate oxidative stress via the Nrf2/HO-1 pathway, and reinforce intestinal epithelial barrier integrity (upregulating tight junction proteins and mucins). Although systematic toxicological characterization remains limited, the generally favorable safety profiles reported in preclinical studies, together with their multi-target nature, suggest that marine polysaccharides hold great potential as functional foods or adjunctive therapies for UC. However, challenges remain in structural heterogeneity, batch-to-batch consistency, and limited clinical validation. Future efforts should prioritize structure–activity relationship elucidation, advanced analytical characterization, bioavailability and gastrointestinal fate studies, standardization and quality control, comparative evaluation against conventional therapies and other natural products, and well-designed clinical trials to facilitate translation from bench to bedside.

## 1. Introduction

Ulcerative colitis (UC), a distinct subtype of inflammatory bowel disease (IBD), has evolved from a relatively rare condition to a global health challenge with accelerating incidence rates in newly industrialized countries [1,2]. Characterized by chronic, relapsing inflammation restricted to the colonic mucosa, the etiology of UC (Figure 1) is multifactorial, involving a complex interplay between genetic susceptibility, environmental triggers, epithelial barrier defects, and dysregulated immune responses against the gut microbiota [3,4,5]. Clinically, patients suffer from debilitating symptoms including bloody diarrhea, abdominal pain, and urgency, which severely compromise quality of life [6,7]. Current pharmacological strategies (Figure 2) primarily aim to induce and maintain remission through 5-aminosalicylates, corticosteroids, immunomodulators, and biological agents (e.g., anti-TNF-α antibodies) [8,9]. While these therapies have transformed patient care, they are far from perfect. A significant proportion of patients experience primary non-response or secondary loss of response to biologics [10,11]. Furthermore, long-term administration of these immunosuppressive agents is frequently associated with severe adverse events, such as opportunistic infections and malignancies, creating an urgent clinical imperative to identify safer, effective, and biocompatible therapeutic alternatives [12,13].

In the search for novel therapeutic agents, natural polysaccharides have garnered substantial attention due to their generally favorable preclinical safety profiles, biodegradability, and diverse biological activities [14,15]. Unlike synthetic drugs, these macromolecules often exert therapeutic effects through a multi-target approach. They not only modulate the host immune system to dampen excessive inflammation but also act as prebiotics to restore intestinal homeostasis [16,17]. Within the gastrointestinal tract, polysaccharides can survive gastric acid digestion to reach the colon, where they are fermented by the commensal microbiota into short-chain fatty acids (SCFAs), such as butyrate and acetate [18,19]. These metabolites play a pivotal role in reinforcing the intestinal epithelial barrier, upregulating the expression of tight junction (TJ) proteins, and inhibiting pro-inflammatory pathways [20,21]. While terrestrial plant-derived polysaccharides have been extensively studied, the vast reservoir of the marine environment offers a unique and structurally distinct class of bioactive carbohydrates that remains underutilized in UC management.

Marine-derived polysaccharides, predominantly isolated from seaweeds (algae), marine animals (shellfish), and marine microorganisms, possess unique structural features that distinguish them from their terrestrial counterparts [22,23]. These structural features, including monosaccharide composition, glycosidic linkages, molecular weight, and sulfation patterns, are systematically summarized in Table 1 and discussed in Section 5. In the context of UC, emerging evidence suggests that these marine macromolecules exhibit potent anti-inflammatory effects by suppressing the NF-κB and MAPK signaling pathways and inhibiting the activation of the NLRP3 inflammasome [24,25]. Moreover, their superior mucoadhesive properties allow for prolonged retention on the inflamed colonic mucosa, potentially forming a protective “bandage” that facilitates tissue repair [26]. The harsh and competitive marine environment has driven the evolution of these complex compounds, endowing them with exceptional antioxidative and immunomodulatory capabilities that are particularly relevant to the pathophysiology of UC.

Despite the promising therapeutic potential of marine-derived polysaccharides, the translation from bench to bedside faces challenges related to structural heterogeneity and the lack of comprehensive mechanistic understanding. The relationship between the specific chemical structures of these polysaccharides (e.g., degree of sulfation, monosaccharide composition) and their anti-colitis efficacy requires systematic elucidation. Therefore, this review provides a comprehensive overview of recent advances in the application of marine-derived polysaccharides for the treatment of colitis. We categorize these polysaccharides based on their origin and structural characteristics and critically examine the molecular mechanisms underlying their protective effects, including mucosal barrier reinforcement, immune modulation, and gut microbiota regulation. Finally, we discuss the current limitations and future perspectives for developing these marine treasures into clinical therapeutics.

## 2. Resources of Marine Polysaccharides Against Colitis

The ocean harbors abundant biological resources and serves as a vast reservoir for the discovery of novel functional molecules against colitis. To date, a wide array of marine-derived polysaccharides (Figure 3 and Figure 4, Table 1) with potential anti-UC activity have been isolated, with sources exhibiting remarkable phylogenetic diversity that can be primarily categorized into algae, animals, and microorganisms.

Among these, macroalgae (seaweeds) represent the most abundant source and can be further classified by their pigmentation. Brown algae-derived polysaccharides, particularly fucoidans, are extensively studied; prominent examples include those from *Sargassum fusiforme* (PSF-T2) [37], *Saccharina japonica* (SDF) [51], *Ishige okamurae* (IOP-0) [53], *Fucus vesiculosus* (FvF) [68], *Macrocystis pyrifera* (DP-MPF) [69], *Scytosiphon lomentaria* (SLF) [70], *Undaria pinnatifida* (UPF) [74], and *Costaria costata* (FCC) [76]. Alginate, another key component from brown seaweed, also demonstrates notable efficacy. Red algae are valuable sources of sulfated polysaccharides and porphyrans, with species such as *Gracilaria birdiae* (GBP) [27], *G. lemaneiformis* (GLP, SP) [32,33,34], *G. caudata* (PLS) [35], and *Pyropia yezoensis* (Porphyran) [67] all demonstrating efficacy. Green algae are equally abundant, providing ulvans and other unique polysaccharides, including BMP from *Blidingia minima* [29], ECP and EDFM from *Enteromorpha clathrata* and *E. prolifera* [42,43,57], and UPP from *Ulva pertusa* [55]. Additionally, microalgae constitute a promising source of polysaccharides, such as ChPS from *Chlorella pyrenoidosa* [54], SpPS from *Spirulina platensis* [54], SyPS from *Synechococcus* sp. [54], CPP from Chrysophyta [58], and PTPs from *Phaeodactylum tricornutum* [60].

Beyond algae, marine animals represent another significant source of anti-colitis polysaccharides. Among echinoderms, sea cucumbers, including HLP from *Holothuria leucospilota* [49], Fuc-Sc from *Stichopus chloronotus* [72], and Aj-FUC from *Apostichopus japonicus* [75], exhibit excellent immunomodulatory activities attributed to their sulfated polysaccharides. Mollusks also contribute notably, with examples including MP from *Mytilus coruscus* [38], JSP and REP from the jellyfish (*Rhopilema esculentum*) [40,45,46], AGSP from the Pacific abalone [41], and OPS from the oyster [47]. Chitosan, derived from the exoskeletons of crustaceans, adds another dimension to this category [65]. Finally, marine microorganisms are also proving to be a valuable source, with examples including YCP from *Phoma herbarum* YS4108 [30] and the extracellular polysaccharide from the fungus *Hansfordia sinuosa* (HPA) [56]. Collectively, this extensive and taxonomically diverse collection of polysaccharides provides a vast natural library for the discovery and development of novel anti-colitis agents.

## 3. Preparation Methods of Marine Polysaccharides Against Colitis

The preparation of marine polysaccharides with therapeutic potential against colitis follows a series of established and methodical steps, typically involving pretreatment, extraction, and purification. While the specific conditions vary depending on the source material, including macroalgae (e.g., *Ulva pertusa* and *Saccharina japonica*), microalgae (e.g., *Chlorella pyrenoidosa*), animals (e.g., oyster and *Stichopus chloronotus*), and microorganisms (*Hansfordia sinuosa*), the core strategies exhibit significant commonality. The primary goal is to isolate bioactive polysaccharides in their native or modified form to ensure high purity and yield for subsequent in vivo evaluation.

The initial step almost invariably involves pretreatment and disruption of the raw material. For algae and animal tissues, this begins with cleaning, drying, and mechanical grinding into a fine powder to increase the surface area for extraction. A crucial preliminary step for many algal and animal samples is the removal of lipids, pigments, and other small molecular interferences using organic solvents. For instance, *E. clathrata* and oyster powders are commonly treated with acetone or 85% ethanol at elevated temperatures (e.g., 75 °C) to eliminate soluble fats, pigments, monosaccharides, and oligosaccharides [44,48]. This ensures that the subsequent extraction yields a polysaccharide fraction with fewer impurities. For fungal sources like *Hansfordia sinuosa*, the process begins with fermentation, where the fungus is cultured in a specific medium before the extracellular polysaccharides are harvested from the broth [56].

Following pretreatment, the most widely adopted technique is hot water extraction. This method leverages the high solubility of polysaccharides in hot aqueous solutions. The parameters, however, are highly source-dependent and are optimized for each material. A review of the provided data reveals a typical temperature range of 80 °C to 100 °C. For example, polysaccharides from the alga *Ishige okamurae* are extracted at 65 °C with CaCl_2_ [53], while those from *Gracilaria lemaneiformis* require 90 °C [32]. Extraction times can vary from 45 min for *Blidingia minima* to 3–6 h for more resilient materials like *E. clathrata* and jellyfish [29,40,44]. The liquid-to-solid ratio is another critical factor, ranging from 1:5 (*w*/*v*) for *Blidingia minima* to a more diluted 40:1 for microalgae like *Chlorella pyrenoidosa* [29,54]. In some cases, enzymatic assistance is employed to improve yield, such as the use of papain for hydrolyzing animal tissues from sea cucumbers (*Holothuria leucospilota* and *Stichopus chloronotus*) or cellulase for breaking down algal cell walls (*Scytosiphon lomentaria*) [50,71,73].

After extraction, the resulting aqueous solution contains a complex mixture of polysaccharides along with proteins and other co-extracted substances. Therefore, a series of purification steps is essential. The most universal method is ethanol precipitation, where ethanol (typically 80% final concentration) is added to the concentrated extract to precipitate the high-molecular-weight polysaccharides, which are then recovered by centrifugation. To obtain a protein-free product, a deproteinization step is critical, most commonly achieved using the Sevage method (a mixture of chloroform and isoamyl alcohol), which denatures proteins and allows them to be separated from the aqueous polysaccharide phase. For higher purity, especially for structural analysis or precise mechanistic studies, chromatographic techniques are employed. This often involves an initial separation on an ion-exchange column (like DEAE-cellulose or Q Sepharose Fast Flow), which separates polysaccharides based on their charge, followed by size-exclusion chromatography (like Sephacryl or Sephadex columns), which fractionates them by molecular weight. This dual-step chromatography is evident in the preparation of homogeneous polysaccharides like REP from *Rhopilema esculentum* and IOP-0 from *Ishige okamurae* [45,46,53]. Finally, the purified polysaccharide solutions are dialyzed to remove salts and small impurities and then lyophilized (freeze-dried) to obtain a stable, dry powder for long-term storage and experimental use.

For studies specifically targeting UC treatment, some protocols also include modifications to the native polysaccharide. For instance, in the case of *Sargassum fusiforme*, the native polysaccharide was subjected to UV/H_2_O_2_ degradation to produce a lower molecular weight fragment (PSF-T2), which may exhibit different bioactivities [37]. This demonstrates that the extraction process can be tailored not only to isolate a compound but also to engineer its physicochemical properties to potentially enhance its therapeutic effects. Collectively, these established and rigorous extraction methods are fundamental to obtaining high-quality marine polysaccharides, providing a solid foundation for investigating their structure and their multifaceted roles in alleviating UC.

For studies specifically targeting UC treatment, some protocols also include modifications to the native polysaccharide. For instance, in the case of Sargassum fusiforme, the native polysaccharide was subjected to UV/H_2_O_2_ degradation to produce a lower molecular weight fragment (PSF-T2), which may exhibit different bioactivities [37]. This demonstrates that the extraction process can be tailored not only to isolate a compound but also to engineer its physicochemical properties to potentially enhance its therapeutic effects. However, extraction and purification procedures should not be regarded as structurally neutral or universally standardized. Differences in extraction conditions (e.g., temperature, time, pH, solvent, ionic strength), deproteinization (e.g., Sevage method), precipitation (e.g., ethanol concentration, CTAB), enzymatic hydrolysis (e.g., enzyme specificity and degree of hydrolysis), oxidative degradation (e.g., UV/H_2_O_2_), and purification (e.g., ion-exchange and size-exclusion chromatography) can alter molecular weight, monosaccharide composition, glycosidic linkages, chain conformation, sulfate content and substitution pattern, branching, and co-purified contaminants, thereby modifying gut microbiota fermentation, barrier protection, immunomodulation, and anti-inflammatory activity. For example, hot water or alkaline extraction may cause depolymerization and sulfate loss; acidic or oxidative treatments can reduce molecular weight while introducing carbonyl/carboxyl groups; deproteinization and ethanol precipitation may cause degradation or selectively enrich certain fractions; enzymatic hydrolysis can generate lower-molecular-weight fragments or bioactive oligosaccharides; and chromatographic purification may enrich or remove specific charge/size fractions. Because these structural features are closely linked to anti-colitis efficacy, preparation-dependent structural changes may contribute to inter-study variability among nominally similar polysaccharides. Collectively, these extraction methods provide a practical foundation for obtaining marine polysaccharides, but detailed reporting of preparation parameters, batch-to-batch structural characterization, and side-by-side activity comparisons of well-defined fractions are needed to distinguish true structure–activity relationships from preparation-induced artifacts [77,78].

## 4. Multiple Mechanisms of Marine Polysaccharides Against Colitis

Based on current studies, the natural polysaccharides from marine organisms exhibit extensive effects in the treatment of UC via a synergistic, multilayered network of mechanisms (Figure 5, Figure 6, Figure 7, Figure 8 and Figure 9, Table 2). Their effects extend beyond a single target, strategically acting at multiple critical stages of colitis pathogenesis, including dampening inflammatory signaling, modulating immune cell activity, strengthening the intestinal epithelial barrier, and promoting a favorable gut microbial composition. These pathways are closely linked and can mutually enhance their actions, ultimately contributing to reduced colonic inflammation and facilitating mucosal repair. The subsequent sections will provide a detailed examination of how marine polysaccharides contribute to these mechanisms, emphasizing their promising potential as a multi-target therapeutic approach for colitis.

To avoid overinterpreting the term “multi-target,” the following sections distinguish five activity domains: (i) prebiotic/microbiota-modulating activity, (ii) anti-inflammatory activity, (iii) immunomodulatory activity, (iv) barrier-restoring activity, and (v) antioxidant activity. These domains are not a single unified mechanism; they are separate outcome categories that may be mechanistically linked. Evidence is described as correlative when only microbial composition, cytokine levels, or pathway phosphorylation/expression were measured; as pathway-engagement when receptor/signaling changes were shown without loss-of-function intervention; and as causal only when inhibitors, agonists, knockout/knockdown, depletion/reconstitution, or FMT experiments directly tested necessity or sufficiency. Unless such causal evidence is available, statements should be read as proposed associations rather than established central mechanisms.

### 4.1. Reshaping Intestinal Microflora

In the following discussion, the terms “beneficial” and “pathogenic” are used operationally to reflect the original authors’ classification or the taxa’s reported positive/negative association with colitis protection. These labels are context-dependent, and relative abundance does not necessarily reflect absolute abundance, metabolic activity, or causal contribution. Similarly, the Firmicutes/Bacteroidota (F/B) ratio is reported where used in the original studies, but it is not a sufficiently robust standalone biomarker of dysbiosis or recovery in UC; it can be influenced by diet, sample processing, sequencing platform, disease stage, and strain-level functional differences, and may mask changes in specific taxa. Therefore, F/B ratio changes are considered supportive, not definitive.

The relationship between the gut microbiota and colitis is characterized by a critical cycle of dysbiosis and drive. In colitis patients, microbial diversity is typically significantly reduced, marked by a decline in beneficial bacteria (such as butyrate-producing species) and an overgrowth of pathogenic ones [79]. This imbalance weakens the intestinal mucosal barrier, allowing bacteria and their metabolites to penetrate the lining and trigger an overactive immune response, which induces persistent colonic inflammation. Consequently, gut dysbiosis serves as both a key link in the initial pathogenesis of colitis and a major driver of its chronic progression and frequent relapses [80].

The gut microbiota ferments dietary fiber to produce SCFAs. SCFAs exert crucial anti-inflammatory and mucosal barrier-protective effects by activating specific receptors (e.g., GPR43) on intestinal and immune cells. In colitis patients, gut microbiota dysbiosis leads to decreased SCFA production and impaired receptor signaling. This disrupts mucosal defense mechanisms and triggers abnormal immune activation, thereby exacerbating intestinal inflammation and tissue damage [81]. In the study by Han et al., they explored the effect and mechanism of sulfated polysaccharide (SP) from *Gracilaria lemaneiformis* against colitis [33]. The results showed that SP significantly modulated the gut microbiota composition, and the abundance changes in key genera such as *Enterorhabdus*, *Desulfovibrio*, *Alistipes*, and *Bacteroides acidifaciens* were closely associated with the strongest protective effects. The SCFAs produced by these gut microbes were efficiently absorbed by the intestinal epithelium, leading to a significant upregulation of the mRNA expression of the SCFA receptors GPR43, Olfr78, and GPR109A in the colon (increased by 0.72, 1.32, and 2.33-fold, respectively, at the highest dose). Additionally, SP suppressed pro-inflammatory cytokines and upregulated TJ proteins and mucins. Thus, the gut microbiota and its downstream SCFA receptors are likely important contributors to the anti-colitis effects of SP, although their causal hierarchy relative to other mechanisms remains to be defined.

In colitis, HIF-1α and the gut microbiota form a bidirectional regulatory axis that maintains intestinal homeostasis. Under physiological conditions, microbial metabolites (such as SCFAs) stabilize HIF-1α, promoting intestinal barrier repair and antimicrobial peptide secretion. In UC, however, dysbiosis exacerbates HIF-1α signaling disruption, leading to thinning of the mucus layer and breakdown of TJs. Targeting HIF-1α stabilization may alleviate colonic inflammation by restoring barrier function and remodeling the microbial community [82]. In the study by Jiang et al., they explored the effect and mechanism of oyster polysaccharides (OPS) against colitis [47]. Experimental evidence suggested that OPS alleviate colitis by regulating the gut microbiota–HIF-1α axis. OPS intervention significantly reduced the disease activity index (DAI) in colitis mice, suppressed the shortening of colon length, decreased MPO activity and the levels of pro-inflammatory cytokines such as TNF-α and IL-6, and increased the anti-inflammatory cytokine IL-10. Mechanistically, OPS acted as a prebiotic that was utilized by the gut microbiota to promote the production of SCFAs including acetate, propionate, and butyrate, while reducing serum lipopolysaccharide (LPS) levels. These SCFAs stabilized HIF-1α protein expression, maintaining intestinal physiological hypoxia, and upregulated its target gene, intestinal trefoil factor. This subsequently restored barrier structures, as evidenced by significantly increased expression of TJ proteins such as ZO-1, Occludin, and Claudin-4, as well as the mucin MUC2, along with recovery of the mucus layer thickness. Correlation analysis revealed that HIF-1α was positively correlated with butyrate, MUC2, ZO-1, and IL-10, and negatively correlated with DAI, MPO, and TNF-α. Collectively, OPS activated the HIF-1α signaling pathway through microbiota-derived SCFAs, thereby rebuilding intestinal barrier homeostasis and suppressing inflammatory responses.

In colitis, gut microbiota dysbiosis disrupts amino acid metabolism, antimicrobial peptide synthesis, and energy metabolism. Microbial imbalance leads to aberrant tryptophan metabolism, resulting in over-activation of the pro-inflammatory serotonin pathway. Meanwhile, reduced levels of amino acids such as arginine and lysine impair antimicrobial peptide synthesis, increasing the risk of pathogenic bacterial colonization. A decrease in butyrate-producing bacteria compromises energy supply to colonic epithelial cells and impairs barrier function. Modulating the gut microbiota can restore these metabolic pathways, thereby alleviating inflammation and repairing the intestinal barrier [83]. In the study by Zhang et al., they explored the effect and mechanism of *Holothuria leucospilota* polysaccharides (HLP) against colitis [49]. Following HLP intervention, colitis rats showed reduced DAI, suppressed colon length shortening, improved histopathological damage, decreased levels of pro-inflammatory cytokines including TNF-α, IL-6, and IL-1β, and increased levels of the anti-inflammatory cytokine IL-10. 16S rRNA sequencing revealed that HLP enhanced gut microbiota diversity, reduced the relative abundance of Firmicutes at the phylum level, increased the abundances of Bacteroidota and Actinobacteria, and enriched beneficial SCFAs-producing genera such as *Lactobacillus* and *Alloprevotella* while reducing opportunistic pathogens such as *Bacteroides* and *Parabacteroides* at the genus level. Metabolomics analysis demonstrated that HLP modulated multiple metabolic pathways, including glutathione (GSH) metabolism, tryptophan metabolism, and methane metabolism. Specifically, HLP influenced amino acid metabolism involving arginine and lysine through regulation of intestinal bacteria such as *Corynebacterium glutamicum* and *Bifidobacterium animalis*, promoted antimicrobial peptide synthesis, and enhanced intestinal barrier function. Additionally, HLP increased butyrate levels, activated energy metabolism in intestinal epithelial cells, and improved the intestinal microenvironment. Correlation analysis revealed significant associations between differential microbiota and metabolites, indicating that HLP alleviated colonic inflammation by regulating the host microbiota cometabolic network and repairing metabolic disorders. In brief, HLP alleviates colitis by remodeling the gut microbiota structure and regulating host metabolism, particularly amino acid metabolism, antimicrobial peptide metabolism, and energy metabolism.

For ECP, the reported increases in Parabacteroides, Alistipes, and Blautia coexisted with a decrease in *Akkermansia muciniphila*. This does not necessarily indicate a detrimental shift. A. muciniphila abundance is sensitive to mucin availability, inflammatory status, and dietary context, and a lower relative abundance may coexist with preserved or enhanced barrier function. The net therapeutic outcome is more likely determined by the integrated microbial community and metabolite profile than by any single taxon; absolute quantification and functional validation are needed before assigning benefit or harm.

The gut microbiota converts primary bile acids into secondary bile acids. In colitis, dysbiosis reduces secondary bile acid production, impairing intestinal immune balance and barrier function, thereby exacerbating colonic inflammation and forming a mutually reinforcing vicious cycle [84]. In the study by Pi et al., they explored the effect and mechanism of alginate from brown seaweed against colitis [66]. The results showed that alginate significantly enriched the abundance of *B. animalis* in the colon (increased from 6.79% to 21.5%) and upregulated bacteria carrying 7α-hydroxysteroid dehydrogenase (7α-HSDH) and bile salt hydrolase (BSH), thereby promoting the synthesis of secondary bile acids. Metabolomics revealed that alginate markedly elevated the level of hyodeoxycholic acid (HDCA), making it the most abundant bile acid in the colon, while inhibiting the phosphorylation of the NF-κB and JNK signaling pathways and reducing the expression of the pro-inflammatory cytokines. The protective effect of alginate was abolished after antibiotic depletion of the gut microbiota, whereas supplementation with either *B. animalis* or HDCA alone recapitulated the anti-inflammatory effects. These findings provide stronger causal evidence for a microbiota–metabolite–effect axis than studies based only on correlative microbiota changes: the protective effect was lost after microbiota depletion and reproduced by *B. animalis* or HDCA. This loss-and-reconstitution design supports, but does not formally prove, that alginate acts through selective microbial enrichment, bile acid remodeling, and subsequent suppression of inflammatory pathways.

Acetovanillone, as a beneficial metabolite, can regulate the gut microbiota structure, promote the metabolism of L-tryptophan into anti-inflammatory products such as indole derivatives, thereby enhancing the intestinal barrier, inhibiting excessive immune activation, and alleviating the inflammatory damage of colitis [85]. In the study by Chen et al., they explored the effect and mechanism of Chrysophyta polysaccharide (CPP) against colitis [58]. The results showed that CPP significantly increased the abundance of beneficial bacteria such as *Akkermansia* and *Bifidobacterium*, while reducing harmful bacteria including *Clostridium sensu stricto 1*, *Escherichia-Shigella*, *Dorea*, and *Parabacteroides*. It also promoted the production of beneficial metabolites, Acetovanillone and L-Tryptophan. These alterations in gut microbiota and metabolites directly inhibited the NF-κB pathway, thereby downregulating pro-inflammatory cytokines, upregulating anti-inflammatory cytokines, and alleviating oxidative stress, which in turn protected the intestinal barrier. In summary, modulation of gut microbiota and their metabolites is proposed to be an important upstream contributor to the anti-colitis effect of CPP; however, causal priority over parallel anti-inflammatory and barrier-protective actions requires further verification.

In the research of Liu et al., they explored the effect and mechanism of *Fucus vesiculosus* fucoidan (FvF) against colitis [68]. 16S sequencing showed that FvF significantly increased the relative abundance of Lachnospiraceae family members (such as *Turicibacter*, *Muribaculum*, *Parasutterella*, and *Colidextribacter*) and promoted the production of SCFAs, including butyrate. The microbial changes subsequently modulated bile acid metabolism: levels of the FXR antagonist β-muricholic acid (βMCA) were reduced, while levels of the FXR/TGR5 agonists cholic acid (CA), deoxycholic acid (DCA), lithocholic acid (LCA), and ursodeoxycholic acid (UDCA) were elevated. These bile acids, acting as endogenous ligands, activate FXR and TGR5 receptors in colonic epithelial cells, enhance intestinal barrier function, and suppress inflammatory responses. Fecal microbiota transplantation experiments showed that protective effects could be transferred to recipient mice using donor microbiota from FvF-treated mice, supporting a contributory role of the gut microbiota–bile acid–FXR/TGR5 axis. However, FMT alone does not exclude the possibility that some microbiota changes are secondary to reduced inflammation; establishing primary causality would require microbiota depletion/reconstitution, selective colonization, or direct metabolite supplementation in the same colitis model.

Based on the study by Xiong et al., the sulfated polysaccharide from *Laminaria japonica* (ALP) exerts protective effects by targeted modulation of gut microbiota structure and their metabolite network [61]. 16S rRNA sequencing revealed that ALP significantly increased the abundance of beneficial genera, including Muribaculaceae, norank_o_Clostridia_UCG-014, and norank_o_RF39, while suppressing the overgrowth of opportunistic pathogens such as Enterobacteriaceae (a major source of LPS) and *Bacteroides*. LEfSe analysis further identified these genera as core biomarkers in the ALP-treated group. Metabolomic analysis showed that ALP significantly elevated fecal concentrations of SCFAs, specifically butyrate, acetate, and propionate. Among these, butyrate serves as a key effector molecule by providing energy for colonic epithelial cells and regulating immune signaling, thereby directly mediating the causal link between microbiota remodeling and colitis remission.

According to the work of Liu et al., *Undaria pinnatifida* polysaccharide (UP) exerts its protective effects primarily by modulating the gut microbiota structure [63]. 16S rRNA sequencing revealed that UP intervention significantly increased the abundance of beneficial genera, including *Akkermansia*, *Parabacteroides*, and *Turicibacter*, while reducing the proportion of potential pathogenic genera such as *Escherichia* and *Clostridium*. Additionally, UP enhanced gut microbiota α-diversity (Shannon index, Simpson index), reduced the Firmicutes/Bacteroidetes (F/B) ratio, and inhibited the overgrowth of Proteobacteria. These findings demonstrate that UP alleviates DSS-induced colitis by remodeling the gut microbiota composition and correcting microbial dysbiosis.

In summary, remodeling the gut microbiota and its metabolites is a central mechanism by which marine-derived polysaccharides alleviate UC. These polysaccharides enrich beneficial bacteria and promote protective metabolites such as SCFAs and secondary bile acids (Figure 5).

### 4.2. Suppression of Inflammation

Throughout this section, changes in phosphorylation or expression of NF-κB, MAPK, AKT, JNK, PPAR-γ, and NLRP3 are interpreted as pathway engagement. Such changes indicate association with anti-inflammatory effects, but they do not by themselves demonstrate causal dependency unless the original study used inhibitors, agonists, genetic knockout/knockdown, or rescue experiments. Therefore, terms such as “suppresses” or “inhibits” reflect the original authors’ interpretation and are not intended to imply causality when only correlative biochemical data are available.

Colitis is essentially a chronic, non-specific inflammation of the colonic mucosa triggered by a combination of factors. Inflammation serves as both the central pathological feature and the direct cause of clinical symptoms like diarrhea, mucoid stools, and abdominal pain [86]. During the disease process, inflammatory responses and tissue damage often form a vicious cycle: continuous infiltration of inflammatory cells destroys the mucosal barrier and creates ulcers, while this damage makes the gut more vulnerable to further irritation, exacerbating the inflammation [87]. Uncontrolled long-term chronic inflammation not only impairs intestinal function but also represents the primary risk factor for colorectal cancer.

In colitis, the AKT and NF-κB signaling pathways are often aberrantly activated. AKT, as an upstream regulator, can activate NF-κB, which then translocates into the nucleus and initiates the transcription of pro-inflammatory cytokines (such as TNF-α and IL-1β), exacerbating the inflammatory response and disrupting the intestinal mucosal barrier. Therefore, the activation of these two pathways is a key mechanism in the pathogenesis of UC [88]. In the study by Song et al., they explored the effect and mechanism of *Blidingia minima* polysaccharides (BMP) against colitis [29]. In a mouse model of DSS-induced colitis, BMP treatment significantly reduced the protein expression levels of phosphorylated NF-κB, IκB-α, and AKT in colon tissue, decreased the mRNA expression and secretion of the pro-inflammatory cytokines IL-1β and TNF-α, and restored the levels of the anti-inflammatory cytokine IL-10. In addition, BMP reduced the levels of myeloperoxidase (MPO) and eosinophil peroxidase (EPO) in the colon, alleviated inflammatory cell infiltration, and restored the expression of the TJ proteins, thereby repairing intestinal barrier function. These results indicate that BMP primarily alleviates colitis symptoms by modulating the NF-κB and AKT pathways to suppress the inflammatory response.

PPAR-γ is a key nuclear receptor that regulates anti-inflammatory responses. Its activation inhibits the NF-κB signaling pathway, thereby reducing the release of pro-inflammatory cytokines and maintaining intestinal immune homeostasis. In colitis, downregulation of PPAR-γ expression leads to failed negative regulation of NF-κB, resulting in excessive activation of NF-κB that drives persistent intestinal inflammation and mucosal damage. Therefore, targeting PPAR-γ activation to block NF-κB-mediated inflammatory cascades represents an important therapeutic strategy for alleviating colitis [89]. In the study by Cao et al., they explored the effect and mechanism of *Saccharina japonica* cellulose (SJC) against colitis [51]. The results showed that in the DSS-induced colitis mouse model, cellulose intervention significantly reduced the DAI, histopathological score, and spleen index. At the molecular level, cellulose significantly down-regulated the mRNA and protein expression of the pro-inflammatory cytokine TNF-α in colon tissue, while significantly up-regulating the expression of the anti-inflammatory factors PPAR-γ and IL-10. Therefore, the inhibition of inflammation plays an important role in the treatment of colitis with SJC.

The MAPK and NF-κB pathways are core drivers of inflammation in colitis. Under colitic conditions, both pathways are aberrantly activated. MAPK (including p38 and ERK) acts in synergy with NF-κB to promote the excessive release of pro-inflammatory cytokines such as TNF-α and IL-1β, thereby exacerbating intestinal mucosal damage and inflammatory infiltration. Together, they constitute a critical inflammatory signaling network [90]. In the study by Son et al., they probed the effect and mechanism of *Ulva pertusa* polysaccharide (UPP) against colitis [55]. Experimental results showed that UPP significantly reduced the levels of p-ERK and p-p38 in colon tissue, blocking MAPK pathway activation, while also inhibiting the degradation of IκBα and preventing NF-κB nuclear translocation. Pathway inhibition further downregulated the expression of multiple pro-inflammatory mediators, including the mRNA level of iNOS, as well as the concentrations of TNF-α, IL-1β, and IL-6 in both serum and colon tissue. Additionally, UPP significantly elevated the levels of anti-inflammatory cytokines IL-4, IL-10, and IgA, while reducing MPO activity, effectively alleviating neutrophil infiltration. These anti-inflammatory effects synergistically improved intestinal barrier function and gut microbiota composition. Collectively, simultaneous inhibition of the MAPK and NF-κB signaling pathways is a major proposed anti-inflammatory mechanism of UPP in colitis.

The NLRP3 inflammasome is a key driver of colitis. In the colonic inflammatory environment, NLRP3 is aberrantly activated, assembling into an inflammasome complex that promotes the maturation and release of pro-inflammatory cytokines such as IL-1β, thereby exacerbating intestinal inflammation and mucosal damage [91]. In the study by Wang et al., they probed the effect and mechanism of *Hansfordia sinuosae* extracellular polysaccharide (HPA) against colitis [56]. Experimental results showed that HPA significantly downregulated the protein expression levels of NLRP3, ASC, and Caspase 1 in colon tissue, thereby blocking the assembly and activation of the inflammasome. Meanwhile, following HPA intervention, the concentrations of the pro-inflammatory cytokines TNF-α and IL-1β in the colon were markedly reduced, while the level of the anti-inflammatory cytokine IL-10 was significantly increased. Notably, this anti-inflammatory effect extended to the central nervous system, as HPA also suppressed the expression of NLRP3 pathway-related proteins in brain tissue and reduced the activation of microglia and astrocytes, thereby ameliorating the depression like behavior associated with colitis. In addition, HPA synergistically repaired the intestinal barrier and modulated the gut microbiota. Thus, inhibition of the NLRP3 inflammasome pathway is a principal proposed anti-inflammatory mechanism of HPA in colitis.

In colitis, the JNK and NF-κB inflammatory pathways are often aberrantly activated. JNK upregulates inflammatory factors through phosphorylation, while NF-κB acts as a key transcription factor directly driving the expression of pro-inflammatory mediators. Their interplay forms a vicious cycle that fuels excessive intestinal immune responses and mucosal damage, representing a core mechanism of disease pathogenesis and a potential therapeutic target [92]. In the study by Pi et al., they explored the effect and mechanism of alginate from brown seaweed against colitis [66]. The results showed that after alginate intervention, the mRNA and protein levels of the proinflammatory cytokines TNF-α, IL-1β, and IL-6 in colon tissue were significantly decreased, and the phosphorylation of NF-κB p65 and JNK was inhibited. Additionally, alginate upregulated the expression of the bile acid receptor FXR in the colon, thereby suppressing the inflammatory response through the FXR-mediated signaling pathway. Therefore, the anti-inflammatory effect of alginate plays an important role in its treatment of colitis.

In the work by Xiong et al., the sulfated polysaccharide from *Laminaria japonica* (ALP) exerts protective effects through a well-defined anti-inflammatory mechanism [61]. ALP intervention significantly reduced the serum levels of key pro-inflammatory cytokines, including TNF-α, IL-6, and IL-1β. Meanwhile, LBP, a biomarker of endotoxemia, was also markedly decreased, indicating that ALP effectively suppresses systemic inflammatory responses. At the colonic tissue level, ALP significantly downregulated the mRNA expression of pro-inflammatory genes (*Tnf*, *Il6*, *Il1b*, and *Nos2*), neutrophil-related chemokines (*Cxcl1*, *Cxcl2*, and *Cxcl3*), and damage-associated molecules (*S100a8* and *S100a9*). Transcriptomic KEGG pathway enrichment analysis further confirmed that the targets of ALP are concentrated in classic inflammatory pathways such as cytokine-cytokine receptor interaction, chemokine signaling pathway, and intestinal immune network. Collectively, ALP inhibits the inflammatory cascade at multiple levels, from systemic circulation to local tissues, representing a key mechanism by which it alleviates UC.

In summary, suppression of colonic inflammation is a central mechanism by which marine-derived polysaccharides alleviate UC. They inhibit key inflammatory pathways such as NF-κB, MAPK, AKT/JNK, and the NLRP3 inflammasome, reduce pro-inflammatory cytokines, upregulate anti-inflammatory mediators, and activate the anti-inflammatory nuclear receptors PPAR-γ and FXR, thereby attenuating mucosal damage and restoring intestinal homeostasis (Figure 6).

### 4.3. Regulation of Immune Response

The core pathogenesis of colitis is fundamentally rooted in the dysregulation of the intestinal immune system. In susceptible individuals, the immune system misinterprets the gut microenvironment, erroneously identifying harmless commensal bacteria or the body’s own tissues as hostile threats [93]. This triggers a cascade of exaggerated immune responses, resulting in the sustained release of pro-inflammatory cytokines. These molecules actively attack and degrade the intestinal mucosal barrier, manifesting as chronic inflammation and painful ulceration [94]. This profound imbalance between immune attack and defense is not merely the catalyst for the disease’s onset, but remains the fundamental reason why colitis often becomes a lifelong, relapsing condition.

The CCL25/CCR9 axis regulates immune cell homing to the gut. In colitis, this axis is overactivated, recruiting pro-inflammatory T cells to infiltrate the mucosa, releasing inflammatory cytokines, and exacerbating mucosal damage, making it a potential therapeutic target [95]. In the study of Lu et al., GLP significantly inhibited the expression of the chemokine CCL25 and its receptor CCR9 in colon tissue, thereby reducing abnormal lymphocyte homing and infiltration into the intestine; simultaneously, it upregulated the levels of the co-stimulatory molecule CD40 and the immunoregulatory factor TGF-β1, promoting the restoration of intestinal immune homeostasis [34]. These findings suggest that CCL25/CCR9-mediated immune homing is a contributory mechanism in GLP treatment of UC.

In colitis, dendritic cells (DCs) and macrophages form a critical immune hub. Both are often overactivated and lose normal tolerance to the gut microbiota. DCs present antigens and drive T cells to differentiate into pro-inflammatory Th1 and Th17 subsets, while macrophages infiltrate in large numbers and polarize toward the M1 phenotype, secreting TNF-α and IL-1β. Together, they amplify the inflammatory cascade, leading to sustained disruption of the intestinal mucosal barrier [96]. In the study by Park et al., they explored the effect and mechanism of *Pyropia yezoensis*-derived porphyran (PYP) against colitis [67]. In both acute and chronic colitis models induced by DSS, oral administration of PYP significantly alleviated body weight loss and colon shortening in mice, and reduced the DAI and histopathological scores. Mechanistic studies revealed that PYP decreased the infiltration of DCs, macrophages, T cells, natural killer cells, and neutrophils in colon tissue, while also suppressing the upregulation of the costimulatory molecules CD40, CD80, and CD86 on DCs and macrophages. At the cytokine level, PYP significantly reduced the levels of the proinflammatory cytokines IFN-γ, IL-17, IL-1β, IL-6, IL-12, and IL-23 in both serum and colon, and downregulated the expression of the transcription factors T-bet and RORγt. Further investigation reported that PYP interacts with TLR2, TLR4, TLR5, and TLR9, thereby blocking dendritic cell activation induced by pathogen-associated molecular patterns and damage-associated molecular patterns, while exerting no direct inhibitory effect on T cells themselves. However, because the summarized evidence does not specify the binding assay (e.g., SPR, ITC, and pull-down) or quantitative affinity parameters, this should be distinguished from simple modulation of TLR signaling; direct binding requires confirmation with quantitative biophysical assays. In conclusion, a major proposed mechanism by which PYP alleviates colitis is targeting DCs and macrophages, inhibiting their activation and antigen-presenting function, thereby indirectly blocking T cell-mediated intestinal immune responses.

Peyer’s patches serve as the central hub of the intestinal mucosal immune system. In colitis, their dysfunction leads to aberrant lymphocyte homing to the intestine, triggering excessive immune responses and persistent inflammation. As a critical target of immune regulation, their status directly determines the direction of intestinal inflammation and the integrity of the mucosal barrier [97]. In the study by Li et al., they explored the effect and mechanism of *Undaria pinnatifida*-derived fucoidan (UPF) against colitis [74]. The core mechanism by which UPF treats colitis lies in its precise regulation of the intestinal immune system, with the modulation of immune responses serving as the decisive therapeutic step. Within Peyer’s patches, the critical inductive sites of gut immunity, UPF significantly downregulates the expression of integrin α4β7 and its ligand MADCAM-1, while simultaneously inhibiting the CCL25 and CCR9 chemokine signaling axis, thereby effectively blocking the aberrant homing of lymphocytes to inflamed intestinal tissues and curbing the excessive recruitment of immune cells to the intestinal mucosa at the source. At the level of the cytokine network, UPF potently suppresses pro-inflammatory cytokines including IL-1β, IL-6, TNF-α, and IFN-γ secreted by activated immune cells, while markedly elevating the levels of anti-inflammatory cytokines such as IL-10 and TGF-β, orchestrating a pivotal shift from a pro-inflammatory to an anti-inflammatory immune state. This immune remodeling effect, which targets Peyer’s patches and focuses on lymphocyte trafficking and cytokine balance, is proposed as a major molecular basis for UPF’s therapeutic action against colitis. The concomitant changes in gut microbiota, including restored microbial diversity, proliferation of reported beneficial bacteria, and enhanced SCFA production, may represent downstream-associated or parallel effects. However, the causal directionality between immune regulation and microbiota/SCFA changes was not definitively established in this study and would require time-course, immune-blockade, or microbiota depletion/reconstitution experiments. In conclusion, this study highlights a potentially important role of Peyer’s patch immune responses in UPF treatment of UC.

In the study of Zhang et al., they explored the effect and mechanism of fucoidan from *Costaria costata* (FCC) against colitis [76]. The underlying molecular mechanism is characterized by FCC markedly inhibiting the infiltration of T cells (particularly CD4^+^ and CD8^+^ T cells) and Ly-6G^+^ neutrophils into colonic tissues. Functionally, FCC blocks helper T cell differentiation, as evidenced by significantly reduced secretion of IFN-γ and IL-17 in colonic CD4^+^ T cells, along with decreased mRNA expression of Th1 (T-bet) and Th17 (RORγt) transcription factors. Further mechanistic studies reveal that FCC does not directly suppress T cell differentiation but instead exerts immunosuppressive effects by directly targeting DCs. In DSS-induced colitis mice, FCC significantly reduces the number of CD45^+^CD11c^+^ DCs in mesenteric lymph nodes and downregulates their expression of co-stimulatory molecules (CD40, CD80, and CD86) and MHC molecules. In vitro, FCC treatment markedly inhibits LPS-induced DC activation, reduces the secretion of pro-inflammatory cytokines from DCs, and subsequently suppresses DC-mediated CD4^+^ T cell proliferation and IFN-γ production. Collectively, FCC alleviates UC-associated intestinal inflammation by inhibiting DC activation, thereby indirectly modulating T cell immune responses.

In summary, regulation of intestinal immune responses is essential for the anti-colitis effects of marine-derived polysaccharides. They suppress dendritic cell and macrophage activation, inhibit Th1/Th17 differentiation and aberrant lymphocyte homing, and restore the balance between pro-inflammatory and anti-inflammatory cytokines, thereby breaking the immune–inflammatory cycle and promoting mucosal homeostasis (Figure 7).

### 4.4. Restoration of Intestinal Barrier

The intestinal barrier serves as the first firewall protecting the gut from exogenous pathogens. In colitis, the physical and chemical structures of the mucosal barrier are compromised, leading to increased intestinal permeability. This allows bacteria and toxins within the gut lumen to penetrate the lining, directly activating underlying immune cells and triggering a sustained overactive immune response and inflammatory damage [98]. This breakdown of barrier function is not only an initiating factor of UC but also the core pathological basis for disease relapse and poor healing, ultimately creating a vicious cycle of barrier disruption and worsening inflammation [99].

In the study by Song et al., BMP upregulated the expression of TJ proteins ZO-1, Occludin, and Claudin-1, reduced serum FITC-dextran permeability, DAO activity, and ET-1 levels, and decreased colonic MPO and EPO activities, thereby repairing the intestinal mucosal barrier [29]. These indicated the significant importance of intestinal barrier repair in BMP treating colitis.

In the research of Han et al., SP significantly upregulated the expression of TJ proteins (Claudin-1, ZO-1) and mucin MUC2 in the colon, thereby enhancing the intestinal mechanical barrier. Simultaneously, it reduced serum levels of endotoxin (ET) and LPS-binding protein (LBP), decreased colonic MPO activity, and inhibited the secretion of pro-inflammatory cytokines [32]. Histologically, SP protected crypt structures, goblet cells, and the muscularis mucosae. Therefore, intestinal barrier repair plays an important role in SP treatment of UC.

In the work of Son et al., they probed the effect and mechanism of *Ulva pertusa* polysaccharide (UPP) against colitis [55]. In in vitro experiments, UPP promoted the secretion of MUC2 and TFF3 by LS174T goblet cells, and upregulated the expression of ZO-1 and Occludin in Caco-2 cells, thereby enhancing transepithelial electrical resistance. In in vivo experiments, oral administration of UPP restored the levels of TJ proteins such as Occludin and Claudin-1 in the colon tissue of DSS-induced colitis mice, increased goblet cell count and mucus secretion, and alleviated mucosal damage and immune cell infiltration. Therefore, intestinal barrier repair is of great significance for the therapeutic effect of UPP on colitis.

Myosin light chain kinase (MLCK) regulates cytoskeletal contraction by phosphorylating myosin light chain 2 (MLC2), leading to the opening of TJs and increased intestinal permeability. In colitis, inflammatory cytokines activate the MLCK/MLC pathway, disrupt the intestinal barrier, and promote antigen translocation and a vicious cycle of inflammation [100]. In the study by Jia et al., they probed the effect and mechanism of *Scytosiphon lomentaria* fucoidan (SLF) against colitis [70]. In mice with fiber-deficient diet-exacerbated colitis, SLF significantly reduced serum levels of LPS and diamine oxidase (DAO), indicating restoration of intestinal permeability. Western blot results showed that SLF downregulated the expression of MLCK, MLC2, and its phosphorylated form p-MLC2, thereby inhibiting cytokine-induced cytoskeletal contraction and TJ leakage. At the same time, SLF significantly increased the expression of TJ proteins ZO-1, Occludin, and Claudin-1, and restored goblet cell numbers and mucin secretion, protecting the intestinal mucus layer. These actions collectively reinforce the intestinal epithelial physical barrier and prevent the invasion of harmful substances, representing a key mechanism by which SLF alleviates colitis. Altogether, SLF protects intestinal barrier integrity by inhibiting the MLCK/MLC pathway and upregulating TJ proteins.

Excessive apoptosis of intestinal epithelial cells disrupts TJs, leading to impaired intestinal barrier and increased permeability. Pathogens and antigens easily penetrate the mucosa, triggering immune inflammation, which further promotes apoptosis and forms a vicious cycle, thereby exacerbating mucosal damage and inflammation in colitis [101]. According to the study of Chen et al., medium and high doses of CPP significantly upregulate the expression of TJ proteins (ZO-1, Occludin, and Claudin-1) in the colon, increase the concentration of mucin MUC2 and the number of goblet cells, and inhibit intestinal epithelial cell apoptosis (decreasing Bax, Bad, and Caspase-3 while increasing Bcl-2) [58]. Transmission electron microscopy revealed that CPP restores the DSS-induced disruption of TJs and adherens junctions at the ultrastructural level, as well as the orderly arrangement of microvilli. Furthermore, CPP reduces serum levels of FITC-dextran and DAO activity, decreases intestinal permeability, and thereby effectively maintains the integrity of the intestinal mucosal mechanical barrier. In summary, repairing intestinal barrier function is of great significance for CPP to improve colitis.

In summary, restoring intestinal barrier integrity is a crucial mechanism by which marine-derived polysaccharides alleviate UC. They upregulate tight junction proteins (e.g., ZO-1, occludin, claudins) and mucins, restore goblet cells, reduce epithelial apoptosis and permeability, and inhibit the MLCK/p-MLC2 signaling, thereby limiting bacterial and toxin translocation and breaking the barrier–inflammation vicious cycle (Figure 8).

### 4.5. Inhibition of Oxidative Stress

Oxidative stress serves as a pivotal mechanism driving tissue damage and the amplification of inflammation in colitis. During the progression of the disease, the excessive accumulation of reactive oxygen species (ROS) overwhelms the body’s endogenous antioxidant defenses, leading to cellular lipid peroxidation, protein denaturation, and DNA damage [102]. This oxidative imbalance directly compromises intestinal epithelial cells and dismantles the mucosal barrier, which in turn triggers pro-inflammatory signaling pathways and intensifies the immune response. The resulting vicious cycle between oxidative injury and inflammatory surge is not only the primary driver behind the formation of mucosal ulcers but also a fundamental pathological factor that leads to the chronic persistence and periodic recurrence of the condition [103].

In the study by Dutra et al., they explored the effect and mechanism of *Gracilaria caudata* polysaccharide (PLS) against colitis [35]. PLS significantly reduced the levels of lipid peroxidation product malondialdehyde (MDA) and nitric oxide (NO) metabolites (NO_3_/NO_2_) in colon tissue, restored the content of the endogenous antioxidant GSH, and inhibited the expression of inducible nitric oxide synthase (iNOS), thereby effectively alleviating oxidative and nitrosative stress injury. On this basis, PLS secondarily reduced MPO activity and the release of pro-inflammatory cytokines (IL-1β, TNF-α), and alleviated inflammatory cell infiltration. Therefore, PLS primarily protects colon tissue by enhancing the body’s antioxidant defense and reducing oxidative damage, with anti-inflammatory effects serving as downstream synergistic actions.

In the work of Cao et al., they explored the effect and mechanism of *Rhopilema esculentum* polysaccharide (REP) against colitis [45]. REP significantly reduced MPO activity and NO levels in colon tissue, inhibited the expression of iNOS, and decreased the production of the lipid peroxidation product MDA. At the same time, it increased the activities of total superoxide dismutase (T-SOD), GSH, and catalase (CAT), thereby enhancing the body’s antioxidant defense capacity. Fecal microbiota transplantation experiments confirmed that these antioxidant effects are closely associated with the gut microbiota modulated by REP. Collectively, antioxidation plays an important role in REP treatment of UC.

In colitis, the Nrf2/HO-1 signaling pathway serves as a key endogenous antioxidant defense mechanism. Activation of Nrf2 induces HO-1 expression, scavenges excessive ROS, and suppresses the NF-κB-mediated inflammatory cascade, thereby alleviating intestinal mucosal oxidative damage and barrier dysfunction. Impairment of this pathway exacerbates colitis, while targeted activation of Nrf2/HO-1 holds clear therapeutic potential [104]. In the study by Li et al., they explored the effect and mechanism of fucoidan of *Stichopus chloronotus* (Fuc-Sc) against colitis [72]. In an H_2_O_2_-induced oxidative damage model of Caco-2 cells, Fuc-Sc pretreatment significantly increased SOD activity and GSH levels, while reducing MDA content and lactate dehydrogenase (LDH) release. The protective effects were reversed by the Nrf2 inhibitor ML385, supporting the involvement of the Nrf2/HO-1 pathway. However, because pharmacological inhibitors may have off-target effects, definitive Nrf2 dependency would require genetic knockout/knockdown or rescue experiments. In a DSS-induced mouse colitis model, oral administration of Fuc-Sc similarly upregulated the expression of Nrf2 and its downstream targets HO-1, NQO1, and GPX-1 in colon tissues, restored SOD activity and GSH levels, and decreased MDA and LDH content, thereby alleviating oxidative damage in the colon. Additionally, Fuc-Sc partially improved the expression of TJ proteins (ZO-1, Claudin-1, Occludin) to repair the intestinal barrier, although its anti-inflammatory effects are primarily dependent on the activation of the antioxidant pathway. In conclusion, activation of the Nrf2/HO-1 signaling pathway is a principal proposed molecular mechanism by which Fuc-Sc alleviates oxidative stress in colitis.

In summary, inhibition of oxidative stress is a key mechanism by which marine-derived polysaccharides alleviate UC. They restore endogenous antioxidant defenses (e.g., SOD, CAT, GSH), reduce MDA, NO, and iNOS, and activate the Nrf2/HO-1 pathway, thereby protecting the intestinal epithelium and interrupting the oxidative stress–inflammation cycle (Figure 9).

## 5. Structure–Activity Relationships Between Marine Polysaccharides and Colitis

The therapeutic potential of marine polysaccharides in colitis is intimately linked to their diverse and complex structural features (Figure 4, Table 1). Understanding the structure–activity relationships (SARs) is crucial for the rational design of polysaccharide-based functional foods and therapeutics. The primary structural parameters governing bioactivity include molecular weight, monosaccharide composition, glycosidic linkages, backbone conformation, and the degree and pattern of sulfation.

### 5.1. Molecular Weight

The molecular weight (Mw) of marine polysaccharides significantly influences their physicochemical properties, fermentation characteristics by gut microbiota, and subsequent immunomodulatory effects in colitis. High-Mw polysaccharides often exhibit poor solubility and limited accessibility to intestinal immune cells, whereas low-Mw fractions are generally more soluble and bioavailable.

Several studies have demonstrated that low-Mw polysaccharides possess superior anti-colitis activity. For instance, a low-molecular-weight fucoidan from *Macrocystis pyrifera* (DP-MPF, 5–30 kDa) effectively alleviated DSS-induced colitis by reducing oxidative stress and pro-inflammatory cytokines [69]. Similarly, a functional fucoidan from *Scytosiphon lomentaria* (SLF, 103 and 460 kDa) protected against intestinal barrier destruction and inflammation [70]. Based on a qualitative synthesis of the available literature, a molecular weight range of approximately 10–100 kDa appears to be associated with favorable anti-colitis efficacy in most studies; yet this proposal derives from qualitative comparisons of the included studies, and systematic quantitative comparisons of activity versus molecular weight are currently lacking. Nonetheless, extremely low Mw may lead to rapid absorption and reduced colonic retention, diminishing prebiotic effects. Conversely, high-Mw polysaccharides like the native fucoidan from *Apostichopus japonicus* (Aj-FUC, 774 kDa) also demonstrated significant protective effects by modulating gut microbiota and enhancing TJ proteins, suggesting that other structural features may compensate for high Mw [75]. However, this remains a speculative interpretation based on the available data, and direct evidence for such compensation is still limited. Notably, directly comparing polysaccharides with vastly different molecular weights (ranging from 4.25 kDa to over 2000 kDa) without accounting for differences in solubility, bioavailability, fermentability, and intestinal retention time is methodologically challenging and may not yield pharmacologically equivalent comparisons. It is also important to note that molecular weight reduction is often accompanied by changes in chain conformation, solubility, and even subtle chemical alterations during degradation (e.g., UV/H_2_O_2_ treatment may introduce oxidation products [37]). Therefore, the improved activity of low-Mw fractions cannot be exclusively attributed to size decrease without controlling for other concomitant structural modifications.

### 5.2. Monosaccharide Composition

The monosaccharide composition is a fundamental determinant of marine polysaccharide bioactivity. Fucose, galactose, rhamnose, and mannose are frequently associated with anti-inflammatory and gut barrier-protective functions.

Fucose-rich polysaccharides are the most extensively studied. Fucoidans from various brown algae, such as *Fucus vesiculosus* and *Undaria pinnatifida*, consistently ameliorate colitis by suppressing Th1/Th17 responses and modulating gut microbiota [68,74]. The presence of galactose in porphyran from *Pyropia yezoensis* (PYP) contributes to its immunomodulatory effects by inhibiting dendritic cell activation [67]. Rhamnose-rich polysaccharides, such as ulvans from *Ulva pertusa* (UPP), exhibit anti-colitis activity by enriching SCFAs-producing bacteria and strengthening the intestinal mucus barrier [55]. Additionally, mannose-containing polysaccharides from marine fungi, like *Hansfordia sinuosa*, have been shown to alleviate colitis by inhibiting the NLRP3 inflammasome [56]. In contrast, glucose-rich polysaccharides without sulfate modification often display weaker activity, highlighting the importance of specific monosaccharides in conferring therapeutic benefits. Nonetheless, monosaccharide composition is rarely the sole variable; it co-varies with sulfation pattern, glycosidic linkage, and molecular weight. Thus, causal attribution to a specific sugar residue should be interpreted with caution and ideally verified by side-by-side comparisons of polysaccharides with similar backbones but distinct sugar profiles [77,78].

### 5.3. Glycosidic Linkages and Backbone Structure

The glycosidic linkage pattern and backbone conformation determine the three-dimensional structure of polysaccharides, influencing their recognition by pattern-recognition receptors and their fermentability by gut microbiota.

Polysaccharides with a backbone of alternating α-(1→3) and α-(1→4)-linked L-fucose residues, common in species like *Costaria costata*, exhibit potent anti-inflammatory effects by directly suppressing dendritic cell activation and subsequent T cell differentiation [76]. Similarly, a fucoidan from *Scytosiphon lomentaria* (SLF) with a similar linkage pattern inhibited the TLR4/NF-κB/MLCK pathway, protecting TJ integrity [70]. In contrast, porphyran from *Pyropia yezoensis* (PYP), which contains alternating 3-linked β-D-galactose and 4-linked α-L-galactose-6-sulfate, alleviates acute and chronic colitis by blocking DC activation and reducing Th1/Th17 responses [67]. The (1→4)-linked rhamnose and glucuronic acid backbone of ulvan from *Ulva pertusa* (UPP) is crucial for its prebiotic effects, promoting the growth of *Parabacteroides* spp. and SCFAs production [42]. These findings indicate that specific glycosidic linkages are not merely structural features but are directly responsible for the biological activity of marine polysaccharides in colitis. While these associations strongly suggest that glycosidic linkage architecture contributes to activity, direct evidence from isomeric polysaccharides differing only in linkage type is scarce [77]. Most conclusions are derived from naturally occurring variants that also differ in other structural features, so the linkage-activity relationship remains largely correlative.

### 5.4. Sulfate Content and Substitution Pattern

Sulfate groups are critical functional moieties that confer negative charge and enable interaction with cationic proteins, chemokines, and pathogen-associated molecular patterns. The degree of sulfation (DS) and the position of sulfate esters profoundly influence the anti-inflammatory potency of marine polysaccharides.

Highly sulfated fucoidans generally exhibit stronger anti-colitis effects. For example, a sulfated polysaccharide from *Gracilaria caudata* (PLS, sulfate content ~1%) still showed significant anti-inflammatory action, but fucoidans with >15% sulfate content, such as those from *Fucus vesiculosus*, display more pronounced immunomodulatory activity [35,68]. The substitution pattern is equally important. Sulfation at the C_2_ and C_4_ positions of fucose residues enhances the ability of fucoidan to block selectin-mediated leukocyte recruitment and inhibit NF-κB activation. In porphyran, sulfate groups at the C6 position of galactose are essential for its inhibitory effect on LPS-induced DC activation [67]. Furthermore, sulfate groups may contribute to prebiotic potential because some SCFA-producing bacteria possess sulfatases that can cleave sulfate moieties. However, direct experimental evidence that desulfation alters fermentability, microbial composition, or SCFA production in the same colitis models is currently lacking; therefore, this possibility should be regarded as a hypothesis rather than an established mechanism [105]. Interestingly, desulfation of ulvan significantly reduces its anti-colitis activity, confirming the indispensable role of sulfate groups [55]. Thus, both the content and the precise location of sulfate substitutions are key determinants of the therapeutic efficacy of marine polysaccharides against colitis. Nevertheless, the current literature lacks systematic studies that compare polysaccharides with identical backbone and molecular weight but varying solely in sulfate content or substitution position [77,78]. Most evidence comes from heterologous sources, making it difficult to isolate the independent contribution of sulfation. Hence, the observed trends should be regarded as indicative rather than definitive, and controlled chemical desulfation or regio-selective sulfation experiments on the same polysaccharide are urgently needed to establish robust causality.

### 5.5. Structure–Activity Relationships: Evidence, Controversies, and Mechanisms

Evidence for structure–activity relationships (SARs) should be interpreted within an explicit hierarchy. Correlative evidence refers to co-variation between a structural parameter and microbiota, cytokine, or barrier endpoints; pathway-engagement evidence requires receptor or signaling changes; causal evidence requires loss-of-function, gain-of-function, desulfation, size-fractionation, linkage-defined analogues, or rescue experiments [77,78]. Most current SAR conclusions for marine polysaccharides in colitis are correlative because studies rarely compare polysaccharides that differ in only one structural parameter. Therefore, associations with molecular weight, monosaccharide composition, glycosidic linkage, or sulfation should not be equated with demonstrated causal structure–activity relationships.

Several critical issues complicate the interpretation of SARs for marine polysaccharides in colitis. First, the vast structural heterogeneity, including not only molecular weight and sulfation but also monosaccharide sequence, branching, and chain conformation, means that “fucoidan,” “ulvan,” “porphyran,” and “alginate” are broad classes rather than defined chemical entities. Consequently, comparisons across studies using different sources, extraction protocols, or even batches may not be pharmacologically equivalent, limiting the generalisability of conclusions [77].

Second, deliberate modifications such as acid hydrolysis, enzymatic degradation, or UV/H_2_O_2_ treatment (e.g., PSF-T2) often alter multiple physicochemical traits simultaneously. Oxidative cleavage may introduce carbonyl or carboxyl groups, change chain flexibility, or affect sulphate stability, making it challenging to assign enhanced anti-colitis effects exclusively to reduced molecular weight [37]. Similarly, chemical desulfation can influence solubility and charge density, yet few studies have examined the impact of desulfation on colitis outcomes while keeping the backbone intact [78].

Third, direct comparisons between native and modified derivatives (e.g., partially hydrolysed, desulfated, or acetylated) within the same polysaccharide series are still limited. Only a handful of reports have addressed the activity changes after selective removal of sulphate groups or controlled depolymerisation, and these are often restricted to in vitro assays. Without such paired experiments, the relative contribution of each structural parameter cannot be rigorously dissected [77,78].

Finally, the multitarget nature of these polysaccharides, which affects microbiota, immunity, barrier function, and oxidative stress, means that even if a structural feature correlates with overall efficacy, it may influence only one submechanism. Future research should adopt well-characterised, chemically defined polysaccharide analogues, combined with orthogonal analytical techniques (e.g., NMR, SEC-MALS, LC-MS), to systematically deconvolute the SARs and enable meaningful cross-study comparisons.

### 5.6. Safety, Model Validity, and Translational Considerations

The claim that marine polysaccharides have “favorable safety profiles” should be qualified. In the studies compiled in Table 2, most compounds were administered orally by gavage at doses ranging from approximately 10 to 600 mg/kg in mice or rats, and no mortality or overt toxicity was generally reported. However, dedicated acute and subchronic toxicity studies, hematological indices (e.g., WBC, RBC, Hb, and PLT), hepatic function (ALT, AST, ALP, and TBIL), and renal function (BUN and Cr) were rarely reported. Where measured, these parameters were mostly unchanged, but the absence of reporting cannot be regarded as evidence of safety. In addition, because marine polysaccharides are structurally heterogeneous and may contain co-extracted proteins, endotoxin, heavy metals, or residual solvents, safety should be assessed for each well-characterized batch rather than assumed from natural origin [22,77,106]. Future preclinical studies should include a toxicity arm with 14-day acute and 28–90-day subchronic observation, hematology, serum biochemistry, organ histopathology, and immunotoxicity endpoints. Until such data are available, “favorable safety” should be described as “generally favorable in efficacy-focused preclinical studies, with limited systematic toxicology.”

Similarly, the translational value of the colitis models summarized in Table 2 is model-dependent. The DSS model is the most frequently used and primarily reflects chemical disruption of the epithelial barrier, innate immune activation, and microbiota-dependent inflammation. It captures several epithelial and innate features of human UC, but it lacks the genetic heterogeneity, environmental triggers, adaptive immune memory, and relapsing-remitting course of human UC [1,2,3,4,5,79,80]. TNBS/ethanol-induced colitis is a hapten-driven, Th1/Th17-biased, and often transmural model that better resembles Crohn’s-like inflammation than typical UC. Acetic acid-induced colitis is a direct chemical burn model dominated by acute innate and oxidative responses, with limited immunological specificity. Cell models (e.g., Caco-2, LS174T, RAW264.7, and dendritic cells) are useful for dissecting epithelial and immune signaling but cannot reproduce the complex mucosal ecosystem, microbiome, and multicellular interactions of human UC [93,94]. Therefore, results from DSS, TNBS, acetic acid, and cell models should not be equated with clinical efficacy in UC; they provide mechanistic hypotheses that require validation in human-relevant systems such as patient-derived organoids, immune-cell co-cultures, and clinical samples.

Finally, Table 2 currently serves as a compilation of model-specific findings rather than a systematic cross-model comparison. A model-stratified reading suggests that some mechanisms are relatively reproducible, including inhibition of NF-κB/MAPK signaling, reduction in oxidative stress via Nrf2/HO-1, upregulation of tight junction proteins and mucins, and promotion of SCFA production. In contrast, mechanisms involving dendritic cell and T-cell modulation, CCL25/CCR9-mediated lymphocyte homing, Th1/Th17 polarization, and bile acid–FXR/TGR5 signaling have been demonstrated mainly in selected models and may be model-dependent [66,67,68,74,76]. However, direct comparison is limited by differences in mouse strain, microbiota baseline, DSS/TNBS/acetic acid concentration and cycle, dose, route, treatment duration, and endpoint definitions. Future studies should test the same well-characterized polysaccharide in at least two mechanistically distinct colitis models and in human-relevant in vitro systems, using standardized endpoints for inflammation, barrier function, microbiota, and metabolites. Such cross-model validation would distinguish core mechanisms from model-specific effects and strengthen translational claims.

## 6. Clinical Evidence and Translational Potential

### 6.1. Current Clinical Evidence

To date, the evidence supporting marine-derived polysaccharides in UC is almost entirely preclinical. The studies summarized in Table 2 were performed in DSS-, TNBS-, or acetic acid-induced colitis models and in intestinal/immune cell models [27,28,29,30,31,32,33,34,35,36,37,38,39,40,41,42,43,44,45,46,47,48,49,50,51,52,53,54,55,56,57,58,59,60,61,62,63,64,65,66,67,68,69,70,71,72,73,74,75,76]. These models capture selected epithelial, innate immune, oxidative, and microbiota-dependent features of UC, but they do not reproduce the genetic heterogeneity, environmental triggers, adaptive immune memory, or relapsing-remitting course of human UC [1,2,3,4,5,79,80,93,94]. At present, direct human evidence remains very limited, and no marine-derived polysaccharide has been established as a standard UC therapy in clinical guidelines [1,2,5]. Therefore, current findings should be regarded as mechanistic and preclinical proof-of-concept rather than clinical evidence.

### 6.2. Safety and Translational Feasibility

The safety of marine polysaccharides has generally been reported as favorable in efficacy-focused animal studies, with oral doses commonly ranging from approximately 10 to 600 mg/kg and no overt mortality or toxicity in most reports [27,28,29,30,31,32,33,34,35,36,37,38,39,40,41,42,43,44,45,46,47,48,49,50,51,52,53,54,55,56,57,58,59,60,61,62,63,64,65,66,67,68,69,70,71,72,73,74,75,76]. However, dedicated acute and subchronic toxicology, hematology, serum biochemistry, organ histopathology, and immunotoxicity data are rarely available, as discussed in Section 5.6 [22,77,106]. Because marine polysaccharides may contain co-extracted proteins, endotoxin, heavy metals, or residual solvents, safety must be established for each well-characterized batch. Translational feasibility will also depend on reproducible manufacturing, defined dosage, route of administration, treatment duration, and potential interactions with conventional UC therapies [1,2,5,8,9,10,11,12,13,22,77,106].

### 6.3. Trial Design Considerations

Future clinical development should use standardized patient populations and endpoints, including clinical response, endoscopic improvement, histological remission, fecal calprotectin, and validated quality-of-life measures [1,2,5]. Mechanistic biomarkers related to barrier function, microbiota composition, SCFAs, bile acids, and inflammatory signaling should be embedded in early-phase trials. Dose-finding studies should account for molecular weight, sulfation, and formulation, because these parameters may determine colonic exposure and fermentation [18,19,20,21,77,78]. Given the current evidence gap, marine polysaccharides are more realistically positioned as adjunctive therapies or functional food candidates rather than replacements for established UC pharmacotherapy [1,2,5,8,9,10,11,12,13].

## 7. Bioavailability, Gastrointestinal Stability, and Fate of Polysaccharides

### 7.1. Stability During Gastrointestinal Transit

After oral administration, marine polysaccharides encounter acidic gastric conditions, digestive enzymes, bile salts, and the colonic microbiota. High-molecular-weight and highly sulfated polysaccharides are generally resistant to gastric and small-intestinal digestion and reach the colon largely intact, where they can be fermented [18,19,20,21,77,78]. However, solubility, viscosity, charge density, molecular weight, and glycosidic linkage influence transit, degradation, and colonic retention. Direct measurements of luminal concentration, fecal recovery, and tissue exposure are rarely reported in the current colitis studies, limiting pharmacokinetic interpretation [22,77,106].

### 7.2. Fermentation and Colonic Persistence

In the colon, polysaccharides are fermented by commensal bacteria into SCFAs, including acetate, propionate, and butyrate, and can also influence bile acid metabolism [18,19,20,21,66,68,81,84]. These metabolites reinforce epithelial energy supply, mucus production, TJ integrity, and immune tolerance [18,19,20,21,81,84]. Mucoadhesive properties may prolong retention on inflamed mucosa and contribute to a protective barrier effect [26]. Low-molecular-weight fragments may be absorbed more readily, whereas very low-molecular-weight fractions may be rapidly cleared and lose prebiotic effects; high-molecular-weight polymers may persist longer but have poor solubility and accessibility [77,78].

### 7.3. Direct Versus Microbiota-Mediated Mechanisms

The relative contribution of direct polysaccharide activity versus microbiota-mediated effects remains unresolved. Stronger causal evidence comes from loss-and-reconstitution experiments: alginate lost its protective effect after antibiotic depletion of the microbiota, while Bifidobacterium animalis or hyodeoxycholic acid supplementation reproduced the anti-inflammatory effect [66]. Fecal microbiota transplantation from FvF-treated mice transferred protection, supporting a contributory microbiota–bile acid–FXR/TGR5 axis, although FMT alone cannot exclude secondary microbiota changes [68]. For most other polysaccharides, microbiota, SCFA, and barrier changes are correlative, and direct binding to receptors or epithelial cells has not been rigorously tested [77,78].

### 7.4. Implications for Delivery and Formulation

Colon-targeted delivery systems, such as pH-responsive nanoparticles, hydrogels, microspheres, or enteric coatings, may improve colonic retention and reduce systemic exposure [26]. Formulation should be guided by structural characterization, because extraction, degradation, and purification can alter molecular weight, sulfate content, chain conformation, and fermentability [77,78]. Future studies should measure gastrointestinal fate, colonic concentration, fermentation kinetics, and metabolite production alongside anti-colitis endpoints.

## 8. Standardization, Structural Characterization, and Quality Control

### 8.1. Structural Heterogeneity and Batch-to-Batch Variation

Marine polysaccharides are structurally heterogeneous, and terms such as fucoidan, ulvan, porphyran, and alginate describe broad classes rather than single chemical entities [77,78]. Source species, harvesting season, geographical location, extraction temperature, solvent, pH, deproteinization, precipitation, enzymatic hydrolysis, oxidative degradation, and chromatographic purification can all change molecular weight, monosaccharide composition, glycosidic linkages, sulfate content and substitution pattern, branching, and co-purified contaminants [22,77,78,106]. These variables can, in turn, alter microbiota fermentation, barrier protection, immunomodulation, and anti-inflammatory activity. Batch-to-batch inconsistency is therefore a major barrier to reproducible pharmacology and clinical translation.

### 8.2. Analytical Characterization

A minimum structural package should include molecular weight distribution, monosaccharide composition, linkage analysis, degree and position of sulfation, protein and endotoxin content, and contaminant profiling [77,78,107]. Appropriate methods include high-performance liquid chromatography, size-exclusion chromatography coupled with multi-angle light scattering, gas chromatography-mass spectrometry, nuclear magnetic resonance spectroscopy, mass spectrometry, and ion chromatography [77,78,107]. For sulfated polysaccharides, selective desulfation and regio-selective sulfation controls are needed to distinguish the contribution of sulfate groups from other structural features [77,78].

### 8.3. Quality Control and Reporting Standards

Future studies should report botanical/marine source and taxonomic identification, extraction and purification parameters, batch number, molecular weight distribution, monosaccharide composition, sulfate content, glycosidic linkage data, and contaminant limits [22,77,78,106]. Reference standards and validated analytical protocols would enable cross-study comparison. Without such standardization, nominally similar polysaccharides from different laboratories cannot be considered pharmacologically equivalent, and structure–activity conclusions remain uncertain [77,78].

## 9. Comparison with Conventional Treatments and Other Natural Products

### 9.1. Conventional UC Pharmacotherapy

Current UC management relies on 5-aminosalicylates, corticosteroids, immunomodulators, biologics, and JAK inhibitors [1,2,5,8,9,10,11,12,13]. These agents have demonstrated efficacy in randomized trials, but a substantial proportion of patients show primary non-response or secondary loss of response, and long-term immunosuppression carries risks of infection and malignancy [8,9,10,11,12,13]. Marine polysaccharides are unlikely to replace these therapies in the near term. Their most plausible positioning is as adjunctive agents or functional food ingredients that may improve barrier function, microbiota homeostasis, and low-grade inflammation with a favorable preclinical safety profile [22,23,77,78,106,107].

### 9.2. Other Natural Products

Terrestrial plant polysaccharides, polyphenols, alkaloids, and probiotics/prebiotics have also been explored in UC [14,15,16,17,18,19,20,21,79,80]. Many share mechanisms such as SCFA production, NF-κB/MAPK inhibition, Nrf2 activation, and TJ upregulation. Marine polysaccharides are distinguished by their high sulfate content, unique glycosidic linkages, and mucoadhesive properties [22,23,26,77,78,106,107]. However, they also face greater structural heterogeneity and supply/quality-control challenges than many terrestrial compounds. Direct comparative studies between marine polysaccharides and other natural products are scarce, and head-to-head trials are needed before claims of superiority can be made.

### 9.3. Positioning and Unmet Needs

The key unmet needs are clinical evidence, standardized products, and comparative efficacy data. Marine polysaccharides should be evaluated as part of a multimodal strategy, with clear definitions of patient population, dose, duration, and endpoints [1,2,5]. Preclinical studies should include active comparators such as 5-aminosalicylic acid or biologics where feasible, and should report whether effects are additive, synergistic, or redundant with conventional therapy [8,9,10,11,12,13]. Such comparisons would clarify the real therapeutic niche of marine polysaccharides in UC.

## 10. Conclusions and Perspective

Marine-derived polysaccharides have emerged as a promising class of natural therapeutic agents for the management of colitis. Their advantages lie not only in their generally favorable preclinical safety profiles and biocompatibility, although systematic toxicological data remain limited, but also in their ability to concurrently target multiple facets of colitis pathogenesis. This review systematically consolidates recent evidence demonstrating that these macromolecules, which originate from seaweeds, marine animals, and microorganisms, ameliorate colonic inflammation through a synergistic network of mechanisms. These include the restoration of gut microbiota homeostasis and metabolite profiles (e.g., SCFAs and bile acids), modulation of intestinal immune responses (e.g., inhibition of DC and T cell activation), suppression of key inflammatory pathways (e.g., NF-κB, MAPK, and NLRP3), alleviation of oxidative stress (e.g., Nrf2/HO-1 activation), and reinforcement of the intestinal epithelial barrier (e.g., upregulation of TJ proteins and mucins). The multi-domain nature of these polysaccharides is particularly attractive given the complex and relapsing course of UC, for which single-target therapies often show limited long-term efficacy, although the strength of evidence differs across these domains. Direct clinical evidence remains scarce, and translation is further limited by unresolved questions in bioavailability, gastrointestinal fate, standardization, quality control, and comparative efficacy, as discussed in Section 6, Section 7, Section 8 and Section 9.

Despite these encouraging advances, the translation of marine polysaccharides into clinical therapeutics faces several critical challenges. First, structural heterogeneity remains a major hurdle. Batch-to-batch variations in molecular weight, monosaccharide composition, degree and pattern of sulfation, and glycosidic linkages lead to inconsistent bioactivity, impeding quality control and reproducible pharmacological evaluation. Second, the structure–activity relationships of marine polysaccharides in colitis are still incompletely understood. While certain trends have emerged, such as the importance of an appropriate molecular weight range (10–100 kDa) and a high sulfate content, systematic structure–activity studies using well-characterized, homogeneous polysaccharide fractions are urgently needed. Third, the majority of studies have been conducted in preclinical animal models, primarily the DSS-induced colitis model. Moreover, as discussed in Section 5.5, the confounding effects of multiple structural variables and the lack of systematic modification studies hinder the establishment of unambiguous SARs [77,78]. To overcome these obstacles, future work should prioritise the preparation of structurally defined analogues (e.g., desulfated, size-defined fragments) and employ standardised analytical protocols, allowing direct comparisons between derivatives that differ in only one parameter. Only then can we move from correlative associations to a true mechanistic understanding of how specific structural features drive anti-colitis activity. Large-scale, long-term, and well-designed clinical trials are essential to validate the efficacy and safety of these compounds in human UC patients. Fourth, the optimal route of administration, dosage regimen, and potential synergistic effects with existing UC therapies remain to be explored.

Looking forward, future research should prioritize several directions. The application of advanced analytical techniques, such as high-performance liquid chromatography coupled with mass spectrometry and nuclear magnetic resonance spectroscopy, will facilitate precise structural elucidation and enable robust quality control. Genetic and metabolic engineering of marine microorganisms may offer a sustainable and reproducible source of structurally defined polysaccharides. Furthermore, the development of oral delivery systems, such as colon-targeted nanoparticles or hydrogels, could enhance the bioavailability and colonic retention of these macromolecules. Finally, integrating multi-omics approaches (metagenomics, metabolomics, and transcriptomics) in both preclinical models and clinical samples will deepen our mechanistic understanding and may identify predictive biomarkers of response. With continued multidisciplinary efforts, marine-derived polysaccharides hold substantial promise to be developed as next-generation functional foods or adjunctive therapeutics for colitis.

## Figures and Tables

**Figure 1:**
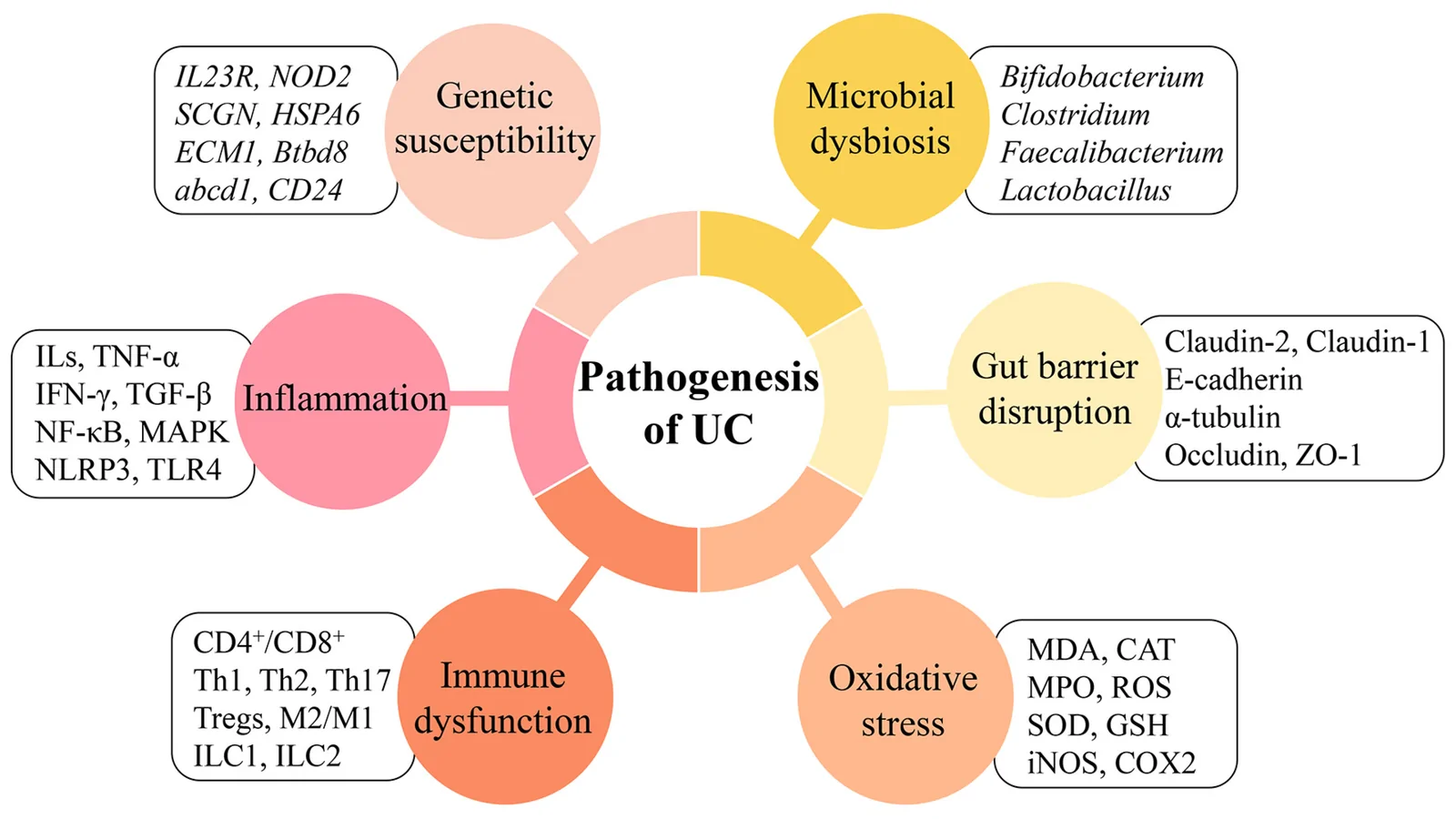
An overview of the underlying pathogenic mechanisms driving ulcerative colitis (UC). The development of UC is driven by a complex interplay of genetic predisposition, immune dysfunction, chronic inflammatory responses, alterations in the gut microbial community, impairment of the intestinal barrier, and excessive oxidative stress.

**Figure 2:**
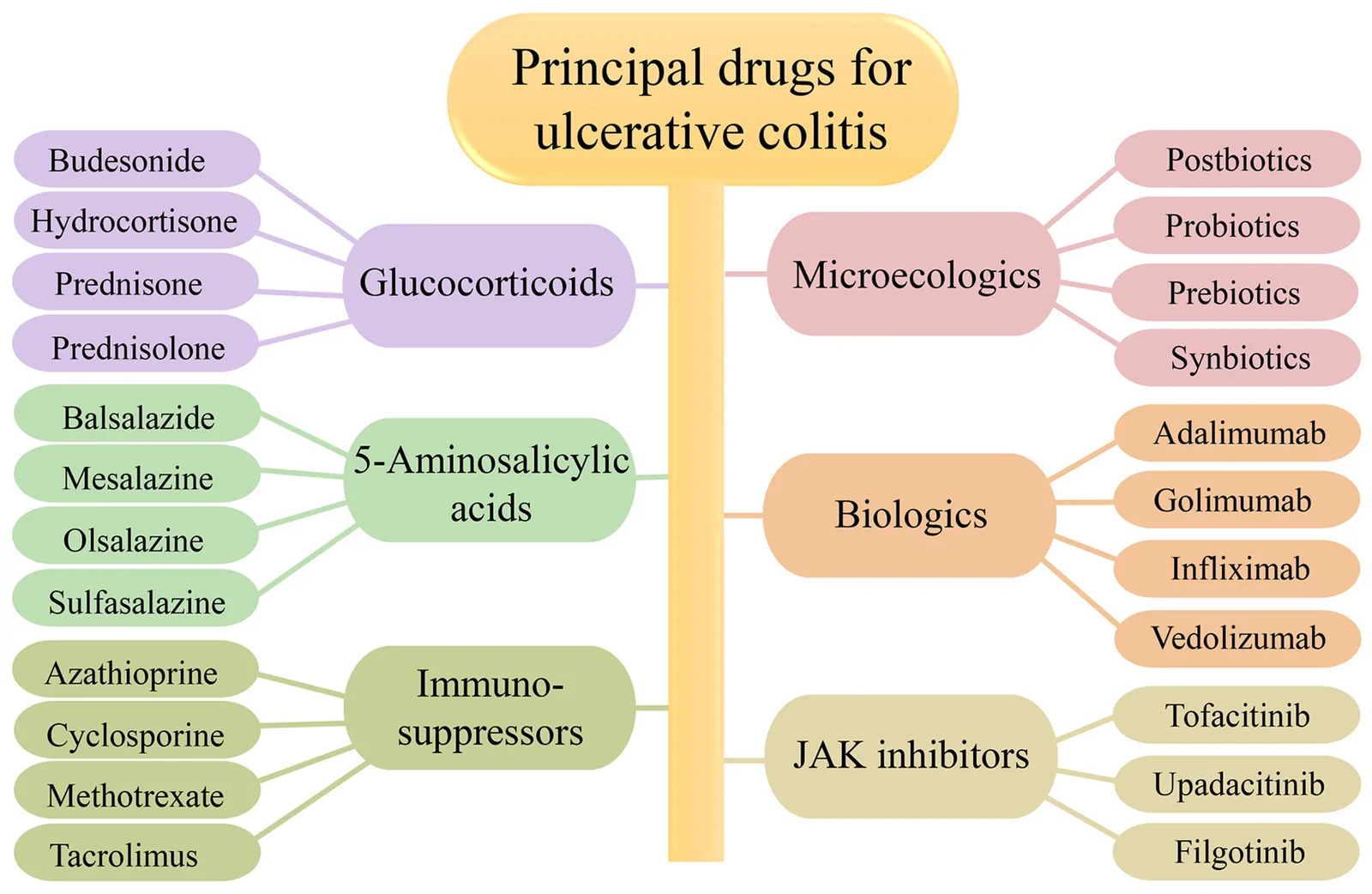
Main medications employed in the treatment of ulcerative colitis (UC). Pharmacological management of UC primarily involves six major therapeutic classes, including 5-aminosalicylic acid derivatives, corticosteroids, immunosuppressive agents, biologic therapies, microecological preparations, and JAK inhibitors.

**Figure 3:**
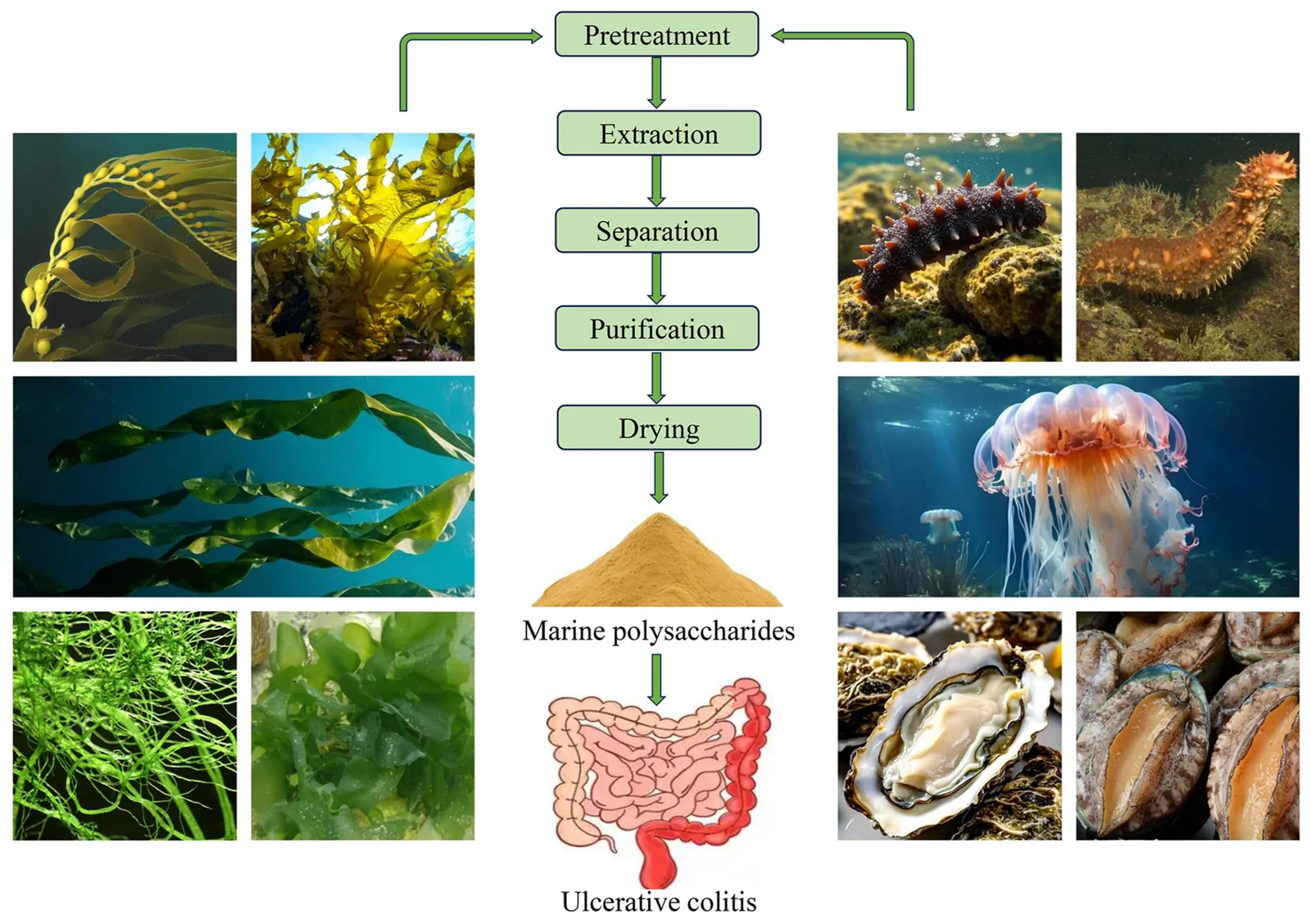
Main sources of marine polysaccharides in the treatment of ulcerative colitis. Resources of marine polysaccharides against colitis mainly include seaweeds (e.g., *Saccharina japonica*, *Undaria pinnatifida*, *Ulva pertusa*, *Gracilaria lemaneiformis*, etc.), marine animals (e.g., Pacific abalone, oyster, jellyfish, *Apostichopus japonicus*, etc.), and marine microorganisms (e.g., *Phoma herbarum* and *Hansfordia sinuosa*).

**Figure 4:**
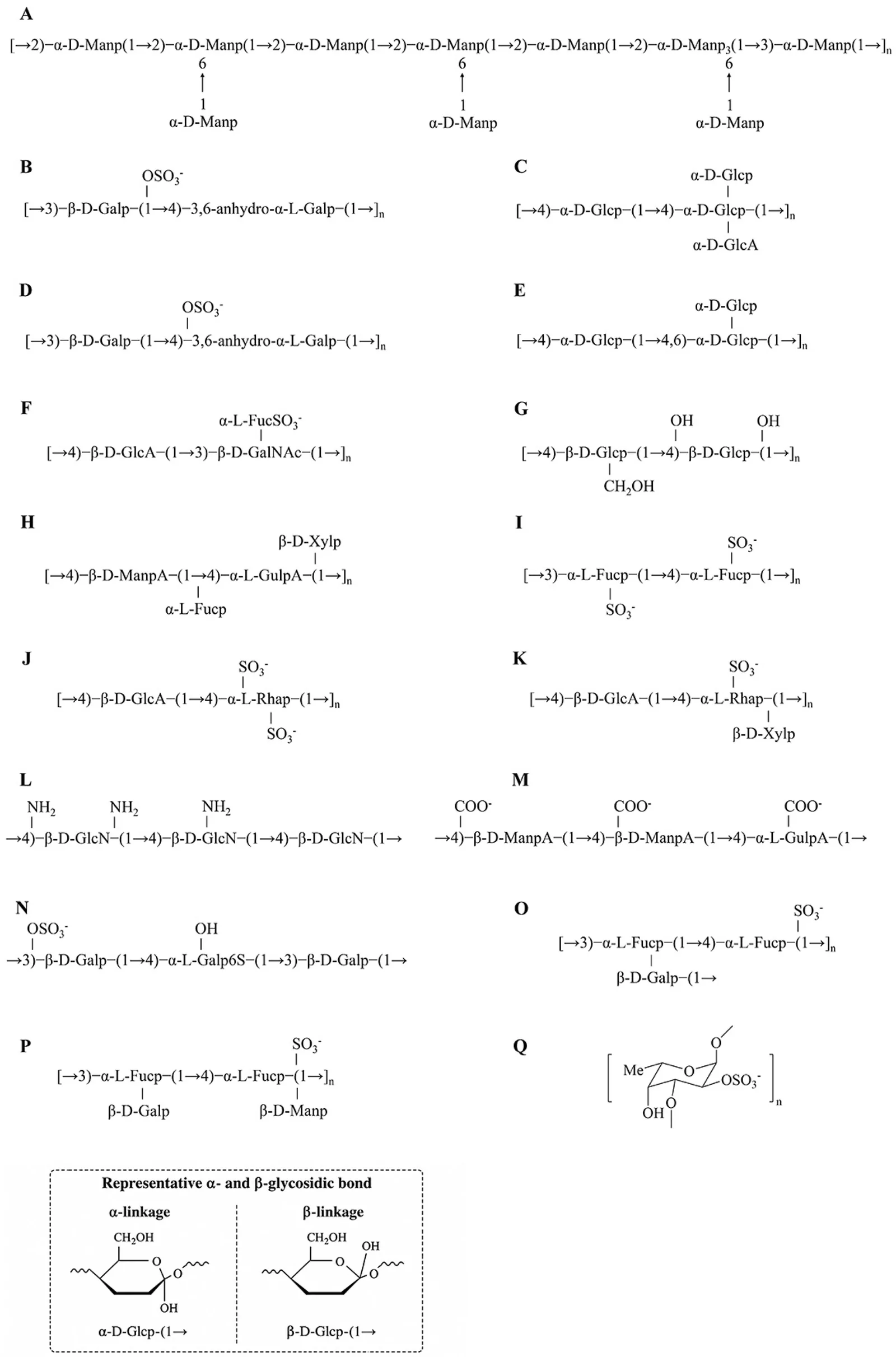
Representative structural models and key features of marine-derived polysaccharides. These models illustrate the primary repeating units and specific functional groups (e.g., glycosidic linkages and sulfation patterns) summarized from the literature, rather than complete molecular structures. (**A**) *Hansfordia sinuosae* mannan (HPA); (**B**) Gracilaria birdiae polysaccharides (GBPs); (**C**) α-D-glucan from *Phoma herbarum* YS4108 (YCP); (**D**) *Gracilaria caudata* polysaccharides (PLSs); (**E**) *Mytilus coruscus* polysaccharides (MP); (**F**) *Holothuria leucospilota* polysaccharides (HLPs); (**G**) *Saccharina japonica* cellulose (SJC); (**H**) dietary fiber from *Saccharina japonica* (SDF); (**I**) *Ishige okamurae* polysaccharides (IOP-0); (**J**) *Ulva pertusa* polysaccharides (UPPs); (**K**) dietary fiber from *Enteromorpha prolifera* (EDFM); (**L**) chitosan from crustaceans; (**M**) alginate from brown seaweeds; (**N**) porphyran from *Pyropia yezoensis*; (**O**) *Macrocystis pyrifera* fucoidan (MPF); (**P**) *Scytosiphon lomentaria* fucoidan (SLF); (**Q**) *Stichopus chloronotus* fucoidan (Fuc-Sc). Inset at bottom: Representative α- and β-glycosidic linkages illustrating the structural difference between α- and β-anomeric configurations.

**Figure 5:**
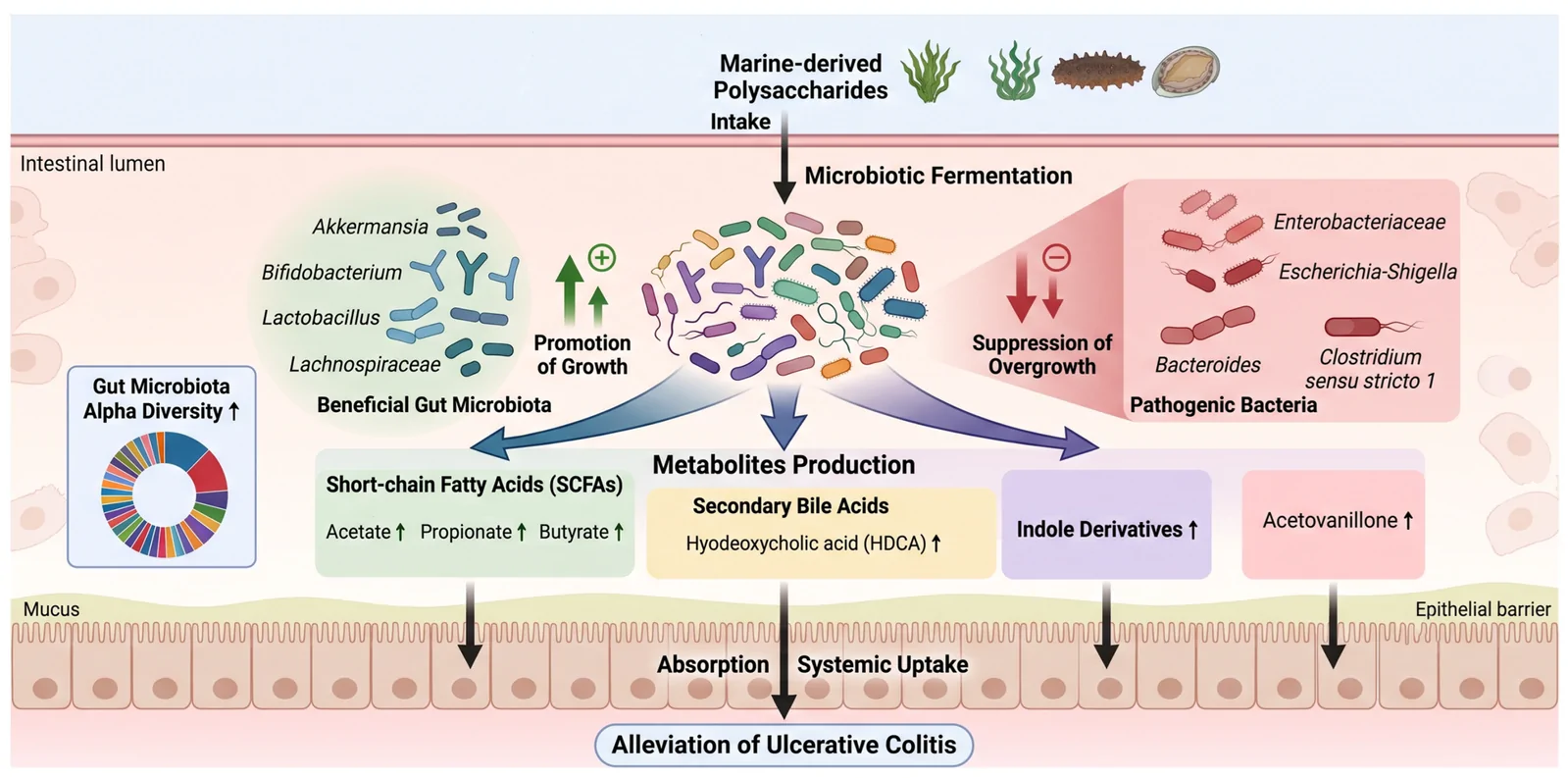
Marine-derived polysaccharides alleviate ulcerative colitis by remodeling gut microbiota and metabolites. Marine-derived polysaccharides reach the colon and are fermented by the gut microbiota. They promote beneficial bacteria (e.g., *Akkermansia*, *Bifidobacterium*, *Lactobacillus*, and Lachnospiraceae) and suppress certain pathogenic/opportunistic bacteria (e.g., Enterobacteriaceae, *Escherichia-Shigella*, *Bacteroides*, and *Clostridium sensu stricto* 1), while increasing alpha diversity. This leads to enhanced production of SCFAs (acetate, propionate, butyrate), secondary bile acids such as HDCA, indole derivatives, and acetovanillone, ultimately contributing to the alleviation of colitis. ↑ indicates an increase; ↓ indicates a decrease.

**Figure 6:**
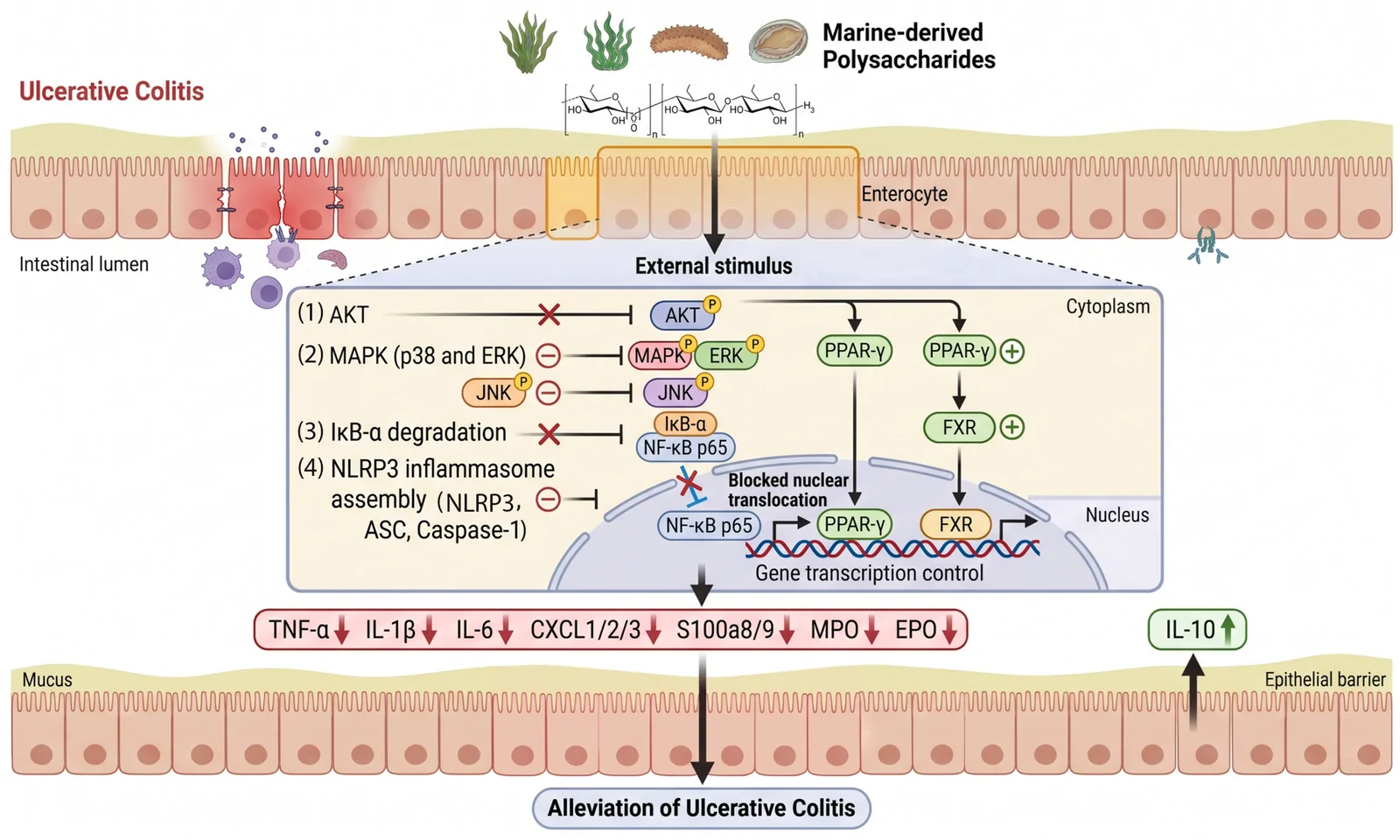
Marine-derived polysaccharides suppress colonic inflammation in ulcerative colitis. Marine-derived polysaccharides inhibit AKT phosphorylation, block IκB-α degradation and NF-κB p65 nuclear translocation, and suppress MAPK (p38 and ERK) and JNK phosphorylation. They also activate PPAR-γ and FXR, and inhibit NLRP3 inflammasome assembly (NLRP3, ASC, and Caspase-1). These actions downregulate pro-inflammatory cytokines (TNF-α, IL-1β, and IL-6), chemokines (CXCL1/2/3), damage-associated molecules (S100a8/9), and enzymes (MPO and EPO), while upregulating IL-10, thereby alleviating colitis. ⊣ indicates inhibition; ↑ indicates an increase; ↓ indicates a decrease.

**Figure 7:**
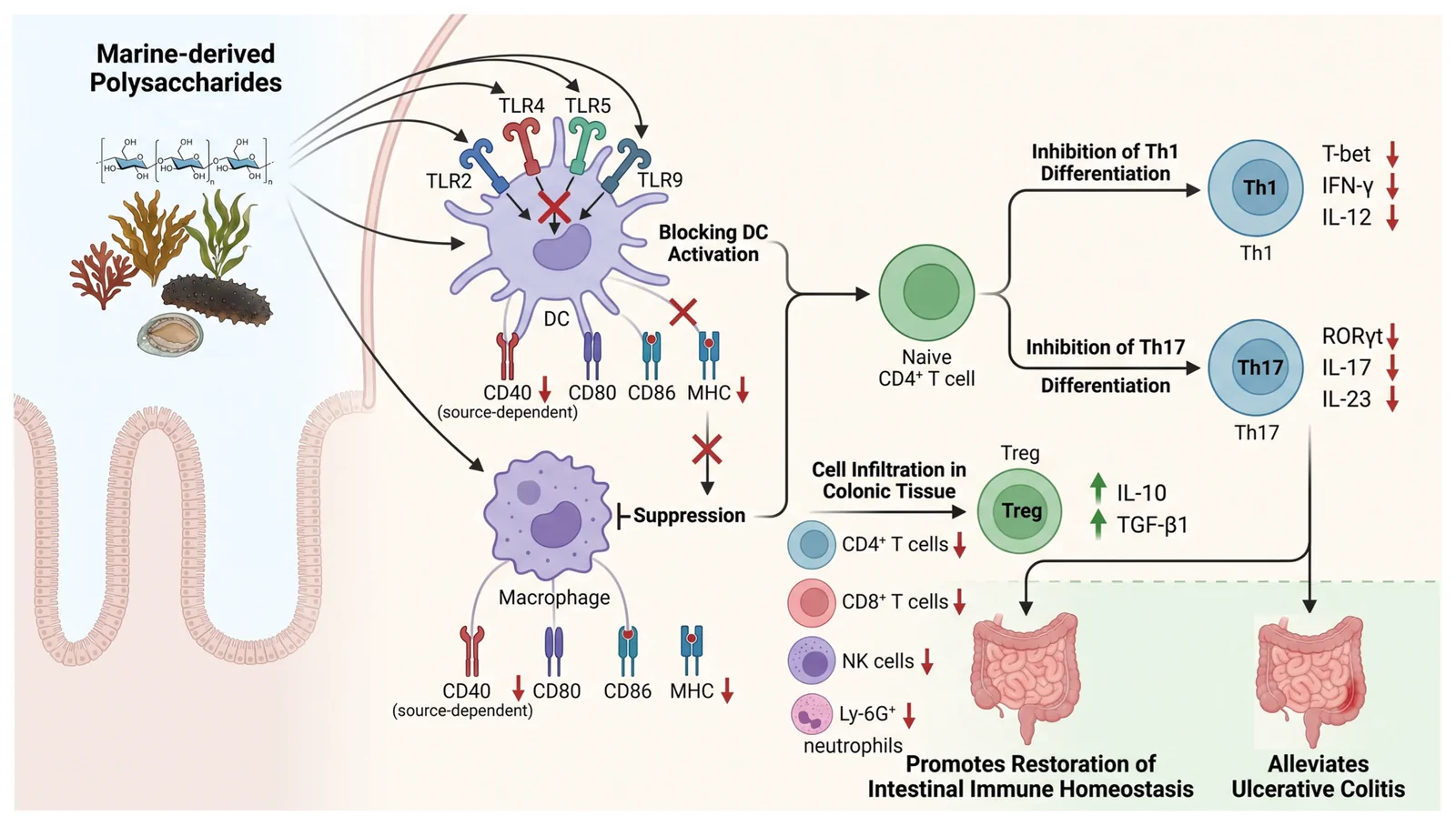
Marine-derived polysaccharides regulate intestinal immune responses in ulcerative colitis. They block TLR2/4/5/9-mediated activation of dendritic cells (DCs) and suppress DC and macrophage activation by downregulating costimulatory molecules (CD40, CD80, CD86) and MHC molecules. They inhibit Th1 and Th17 differentiation, reducing T-bet, RORγt, IFN-γ, IL-17, IL-12, and IL-23, and decrease the infiltration of CD4^+^ T cells, CD8^+^ T cells, NK cells, and Ly-6G^+^ neutrophils. Meanwhile, they upregulate IL-10 and TGF-β1, promoting restoration of intestinal immune homeostasis and alleviating colitis. ↑ indicates an increase; ↓ indicates a decrease.

**Figure 8:**
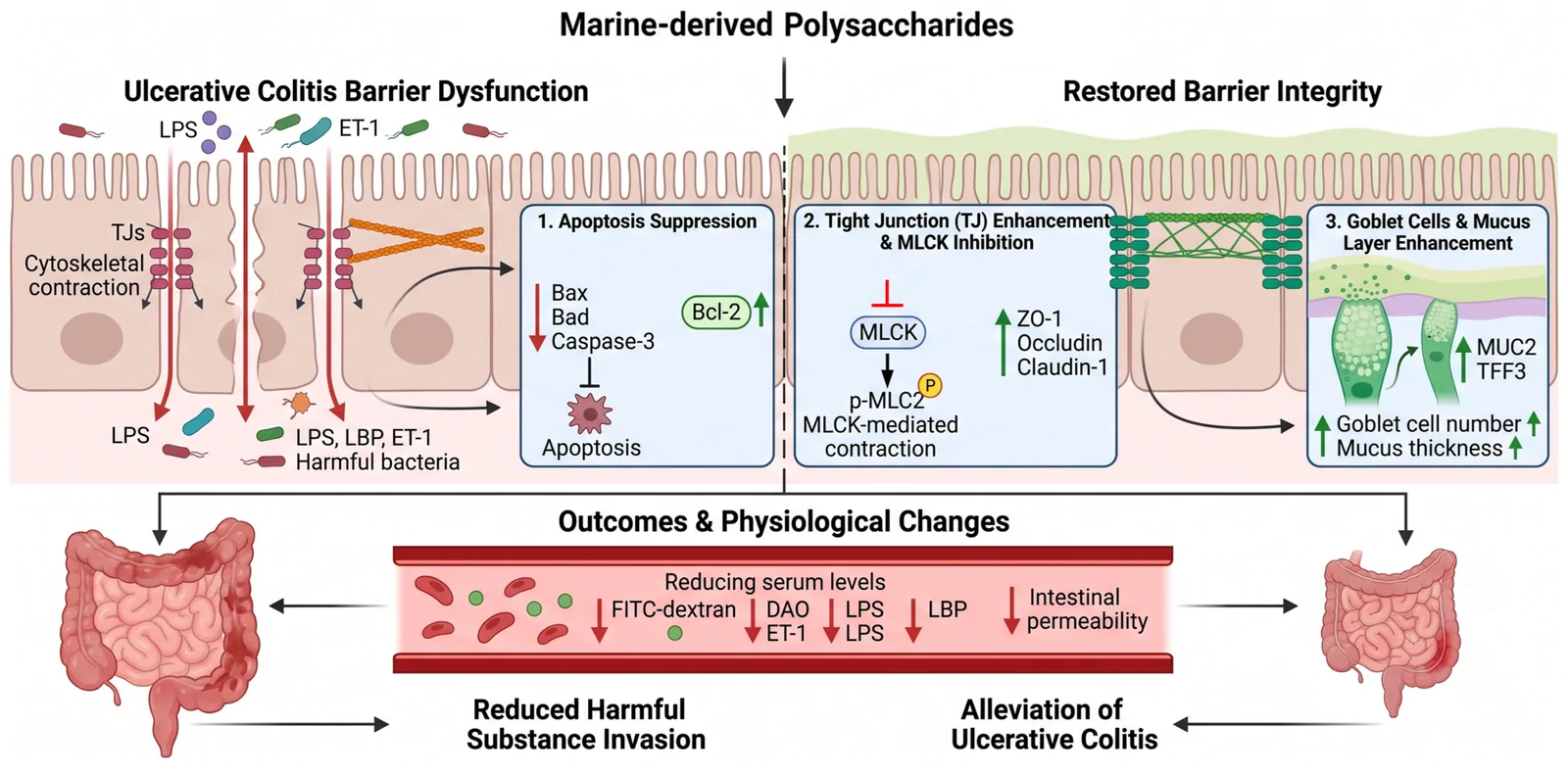
Marine-derived polysaccharides restore intestinal barrier integrity in ulcerative colitis. Marine-derived polysaccharides upregulate tight junction proteins (ZO-1, Occludin, and Claudin-1), promote MUC2 and TFF3 secretion, increase goblet cell number and mucus layer thickness, and enhance transepithelial electrical resistance (TEER). They reduce intestinal permeability, as evidenced by decreased serum FITC-dextran, DAO activity, ET-1, LPS, and LBP levels. Mechanistically, they inhibit the MLCK/MLC2/p-MLC2 pathway, thereby suppressing cytokine-induced cytoskeletal contraction and tight junction leakage. They also inhibit intestinal epithelial cell apoptosis by decreasing Bax, Bad, and Caspase-3 and increasing Bcl-2. These actions collectively reinforce the intestinal epithelial physical and chemical barriers, protecting against harmful substance invasion and alleviating colitis. ⊣ indicates inhibition; ↑ indicates an increase; ↓ indicates a decrease.

**Figure 9:**
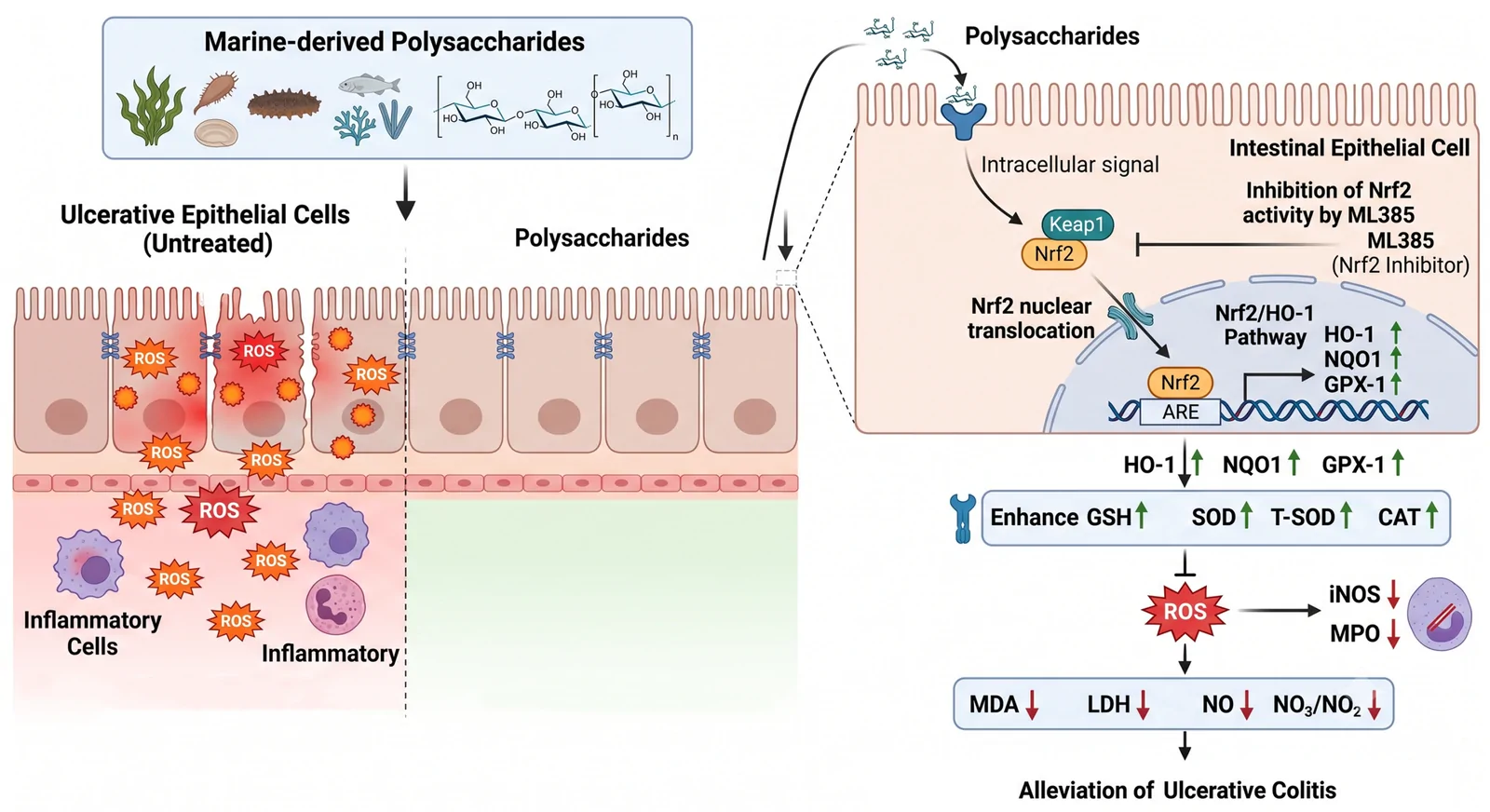
Marine-derived polysaccharides alleviate oxidative stress in ulcerative colitis. Marine-derived polysaccharides enhance endogenous antioxidant defense by increasing GSH levels and SOD, T-SOD, and CAT activities, and by activating the Nrf2/HO-1 pathway, including upregulation of HO-1, NQO1, and GPX-1. They reduce oxidative damage markers such as MDA, LDH, NO, and NO_3_/NO_2_, inhibit iNOS expression, and decrease MPO activity. ⊣ indicates inhibition; ↑ indicates an increase; ↓ indicates a decrease.

**Table 1:** General information on marine-derived polysaccharides against colitis.

Name	Source	Extraction Method	Molecular Weight	Composition	Main Glycosidic Bond	Main Chain	References
GBP	*Gracilaria birdiae*	Hot water extraction, ethanol precipitation, and freeze-drying	370 kDa	Gal (65.4 mol%), AnGal (25.1 mol%), 6-O-Me-Gal (9.2 mol%), 3-O-Me-Gal/4-O-Me-Gal (0.33 mol%)	β-(1→3), α-(1→4)	→4)−3,6-An-α-L-Galp−(1→3)−β-D-Galp−(1→	[27,28]
BMP	*Blidingia minima*	Hot water extraction, macroporous resin adsorption chromatography, ethanol precipitation, and freeze-drying	/	Rha (57.4 mg/g), Xyl (26.9 mg/g), Glc (22.9 mg/g), GlcA (21.9 mg/g), Gal (4.9 mg/g), Man (3.9 mg/g), GalA (4.7 mg/g), GalN (2.4 mg/g), GlcN (1.3 mg/g)	/	/	[29]
YCP	*Phoma herbarum* YS4108	Hot water extraction, deproteinization (Sevage method), dialysis, ethanol precipitation, anion exchange, and gel filtration	2.4 × 10^3^ kDa *	Glc (98%), GlcA (1.5%)	α (1→4)	→4)−α-D-Glcp−(1→	[30,31]
SP	*Gracilaria lemaneiformis*	Citric acid extraction, ethanol precipitation, and freeze-drying	21 kDa	Gal (71.78%), Glc (13.48%)	β-(1→3)	→3)−β-D-Galp−(1→	[32,33]
GLP	*Gracilaria lemaneiformis*	Hot water extraction, ethanol precipitation, and drying	157 kDa	Galp	α-(1→4), β-(1→3)	→4)−α-L-AnGalp−(1→3)−β-D-Galp−(1→	[34]
PLS	*Gracilaria caudata*	Hot water extraction, ethanol precipitation, acetone washing, hot air drying, and secondary ethanol precipitation	116 kDa	Galp	β-(1→3), α-(1→4)	→3)−β-D-Galp−(1→4)−3,6-anhydro-α-L-Galp−(1→	[35,36]
PSF-T2	*Sargassum fusiforme*	Hot water extraction, UV/H_2_O_2_ degradation, dialysis, and freeze-drying	17.31 kDa	Fuc, Gal, GlcA	/	/	[37]
MP	*Mytilus coruscus*	Hot water extraction, deproteinization (Sevage method), ethanol precipitation, anion exchange, and gel filtration	4.25 kDa	Glcp	α-(1→4)	→4)−α-D-Glcp−(1→	[38,39]
JSP	Jellyfish	Hot water extraction, ethanol precipitation, deproteinization, dialysis, and freeze-drying	8.2 kDa	Gal:Glc:Man:Ara = 1.24:1:0.19:0.25	/	/	[40]
AGSP	pacific abalone	Hot water extraction, anion exchange, ethanol precipitation, hydrogen peroxide decolorization, dialysis, and freeze-drying	8 kDa	GlcA, Gal, Man	/	→3)−GlcA−(1→3)−Gal−(1→	[41]
ECP	*Enteromorpha clathrata*	Ethanol degreasing, hot water extraction, ethanol precipitation, dialysis, and freeze-drying	11.67 kDa	Rha (49.7%), Glc (29.9%), GlcA (10.8%), Xyl (7.2%), Man (1.0%), Gal (1.3%)	/	/	[42,43,44]
REP	*Rhopilema esculentum*	Hot water extraction, ethanol precipitation, anion exchange, gel filtration, dialysis, and freeze-drying	10.11 kDa	Man:GlcA:GalN:Glc:Gal:Ara = 0.04:0.12:0.41:1:1.2:0.06	/	/	[45,46]
OPS	Oyster	Acetone degreasing, hot water extraction, deproteinization (Sevage method), ethanol precipitation, dialysis, and freeze-drying	6.83 × 10^3^ kDa	Glc (92.7%), Man (1.85%), GlcN (3.63%), Xyl (2.32%)	/	/	[47,48]
HLP	*Holothuria leucospilota*	Enzymatic extraction, CPC precipitation, ethanol precipitation, decolorization, deproteinization (Sevage method), dialysis, and freeze-drying	106 kDa	GlcA:GalNAc:Fuc = 0.45:1.00:2.40	/	→4)−β-D-GlcA−(1→3)−β-D-GalNAc−(1→	[49,50]
SJC	*Saccharina japonica*	Decalcification, boiling, alkali treatment, drying, crushing, and decolorization	/	Glc	β-(1→4)	→4)−β-D-Glcp−(1→	[51]
SDF	*Saccharina japonica*	Decalcification, boiling, alkali treatment, dialysis, freeze-drying, and concentration	/	ManA, Man, GulA, GlcA, Glc, Xyl, Fuc	/	→4)−β-D-ManpA−(1→4)−α-L-GulpA−(1→	[51,52]
IOP-0	*Ishige okamurae*	CaCl_2_ extraction, ethanol precipitation, anion exchange, gel filtration, and freeze-drying	7.30 kDa	Fuc:Man:Gal:Glc:Xyl = 24.13:2.94:2.74:1.45:1.00	α-(1→3) and α-(1→4)	→3)−α-L-Fucp−(1→ and →4)−α-L-Fucp−(1→ (alternating)	[53]
ChPS	*Chlorella pyrenoidosa*	Hot water extraction, ethanol precipitation, deproteinization (Sevage method), dialysis, secondary ethanol precipitation, and freeze-drying	0.67 × 10^3^ kDa 3.67 × 10^3^ kDa	Rha (38.63 mol%), Glc (6.28 mol%), GlcN (22.13 mol%), GlcA (10.56 mol%), Man (8.02 mol%), Fuc (5.04 mol%), Xyl (3.44 mol%), Gal (5.97 mol%)	→4)-Glcp-(1→ (40.3%) →3)-Rhap-(1→ (20.4%) →3,4)-Rhap-(1→ (16.2%)	→4)−Glcp−(1→ and →3)−Rhap−(1→	[54]
SpPS	*Spirulina platensis*	Hot water extraction, ethanol precipitation, deproteinization (Sevage method), dialysis, secondary ethanol precipitation, and freeze-drying	0.91 × 10^3^ kDa 4.44 × 10^3^ kDa	Rha (33.81 mol%), Glc (34.59 mol%), GlcN (5.83 mol%), GlcA (8.45 mol%), Man (7.85 mol%), Fuc (5.12 mol%), Xyl (4.78 mol%)	→4)-Glcp-(1→ (23.5%) →3)-Rhap-(1→ (9.9%) →3,4)-Rhap-(1→ (9.8%) →2,4)-Rhap-(1→ (8.3%) →3)-Fucp-(1→ (6.9%)	→4)−Glcp−(1→, →3)−Rhap−(1→ and →3)−Fucp−(1→	[54]
SyPS	*Synechococcus* sp. PCC 7002	Hot water extraction, ethanol precipitation, deproteinization (Sevage method), dialysis, secondary ethanol precipitation, and freeze-drying	0.64 × 10^3^ kDa 9.18 × 10^3^ kDa	Rha (19.70 mol%), Glc (5.78 mol%), GlcA (8.74 mol%), Man (37.71 mol%), Fuc (3.60 mol%), Xyl (8.45 mol%), Gal (16.02 mol%)	→4)-Manp-(1→ (23.1%) →2)-Manp-(1→ (11.2%) →3)-Rhap-(1→ (9.2%) →2,6)-Manp-(1→ (8.4%) →3,6)-Galp-(1→ (7.7%)	→4)−Manp−(1→, →2)−Manp−(1→ and →3)−Rhap−(1→	[54]
UPP	*Ulva pertusa*	Hot water extraction, ethanol precipitation, dialysis, and freeze-drying	116.1 kDa	Rha (35.1 mol%), GlcA (39.5 mol%), IdoA (13.2 mol%), Xyl (4.1 mol%), Glc (5.4 mol%), Fuc (2.8 mol%)	α-(1→4), α-(1→3,4), β-(1→4)	→4)−β-D-GlcA−(1→4)−α-L-Rhap−(1→	[55]
HPA	*Hansfordia sinuosae*	Collect fermentation broth, ethanol precipitation, ion exchange, and gel filtration	22.5 kDa	Man (98.2%)	α-(1→4), α-(1→3,4)	→2)−α-D-Manp−(1→ and →3)−α-D-Manp−(1→ (mixed)	[56]
EDFM	*Enteromorpha prolifera*	Hot water extraction and spray drying	410 kDa	Rha:GlcA:Xyl:Glc:Gal:Man:GalA:Fuc = 54.49:24.67:11.97:5.21:2.37:0.60:0.32:0.37	/	→4)−β-D-GlcA−(1→4)−α-L-Rhap−(1→	[57]
CPP	Chrysophyta	Hot water extraction, ethanol precipitation, deproteinization (Sevage method), freeze-drying	259–364 kDa	Glc, Fru	/	/	[58,59]
PTPs	*Phaeodactylum tricornutum*	Ethanol degreasing, hot water extraction, trichloroacetic acid deproteinization, ethanol precipitation, and freeze-drying	12.56 kDa	Rib (50.4%), Rha (15.2%), GlcA (6.3%), Gal (5.4%), GalA (5.0%), Glc (4.4%), Xyl (4.1%)	α-(1→3), β-(1→2,4), β-(1→3), β-(1→5)	→1,3)−α-L-Rhap−(1→, →1,3)−α-L-Fucp−(1→, →1,2,4)−β-D-Galp−(1→, →1,3)−β-D-Xylp−(1→, →1,5)−β-D-Ribp−(1→	[60]
ALP	*Laminaria japonica*	Pretreatment, extraction, filtration and neutralization, concentration and precipitation, centrifugation and collection, redissolution and dialysis, re-concentration and lyophilization	10.69 kDa	ManA (53.33%), GlcA (13.53%), Man (10.50%), Fuc (9.28%), GulA (5.66%)	β-(1→4), α-(1→3), α-(1→4)	→4)−β-D-ManpA−(1→4)−α-L-GulpA−(1→, →3)−α-L-Fucp−(1→, →4)−α-L-Fucp−(1→	[61,62]
UP	*Undaria pinnatifida*	Degreasing and enzymatic extraction, initial centrifugation and precipitation, CTAB precipitation, reconstitution and re-centrifugation, ethanol precipitation and drying	389.2 kDa	Fuc:Rha:Gal:Xyl:Man:GlcUA = 18.98:2.57:75.26:1.00:5.95:11.16	α-(1→3), α-(1→4)	→3)−Fuc(2S)−(1→3)−Gal(4S)−(1→	[63,64]
Chitosan	Crustaceans	/	217 kDa	GlcN	β-(1→4)	→4)−β-D-GlcN−(1→	[65]
Alginate	Brown seaweed	/	21.51 kDa	GulA:ManA = 0.69:1	β-(1→4)	→4)−β-D-ManpA−(1→4)−α-L-GulpA−(1→	[66]
Porphyran	*Pyropia yezoensis*	Hot water extraction, sodium acetate buffering, ethanol impurity removal, ethanol precipitation, dialysis, and freeze-drying	>217 kDa	Gal	α-(1→3), β-(1→4)	→3)−β-D-Galp−(1→4)−α-L-Galp6S−(1→	[67]
FvF	*Fucus vesiculosus*	/	242.24 kDa	Glc:Rha:Fuc:Ara:GlcA:Rib:Xyl:Man = 43:32:11:4:3:3:2:2	α-(1→3), α-(1→4)	α-(1→3)−L-Fucp and α-(1→4)−L-Fucp	[68]
DP-MPF	*Macrocystis pyrifera*	/	17.4 kDa	Fuc (62.9%), Xyl (4.5%), Man (8.1%), Gal (14.3%), Glc (6.9%), Ara (0.4%)	/	→3)−α-L-Fucp−(1→4)−α-L-Fucp−(1→	[69]
SLF	*Scytosiphon lomentaria*	Water extraction, compound enzymatic hydrolysis, ethanol fractional precipitation, deproteinization (Sevage method), and freeze-drying	103, 460 kDa	Man:Gal:Fuc:GalA:Glc:Xyl:Rha:GlcA = 17.1:13.6:15.9:4.7:3.3:5.7:1.0:1.1	/	→3)−α-L-Fucp−(1→4)−α-L-Fucp−(1→	[70,71]
Fuc-Sc	*Stichopus chloronotus*	Papain enzymatic hydrolysis, CPC precipitation, ethanol fractionation precipitation, dialysis, freeze-drying, anion exchange, and gel filtration	779 kDa	Fuc (63.4%)	α-(1→3)	→3)−α-L-Fucp-2S−(1→	[72,73]
UPF	*Undaria pinnatifida*	Ultrasonic extraction, CaCl_2_ precipitation, ethanol precipitation, deproteinization (Sevage method), column chromatography purification, dialysis, and freeze-drying	25.02 kDa	Gal:Fuc = 0.8:1.0	α-(1→3), α-(1→4), β-(1→3)	α-(1→3/1→4)−L-Fucp	[74]
Aj-FUC	*Apostichopus japonicus*	Enzymatic protein release, ethanol precipitation, dialysis, and freeze-drying	774.2 ± 1.34 kDa	Fuc:Glc:Man:GalN:Gal:GlcN = 51:20:15:6:4:4	α-(1→3)	→3)−α-L-Fucp−(1→	[75]
FCC	*Costaria costata*	Ethanol protein removal, hot water extraction, CaCl_2_ precipitation, graded alcohol precipitation, HCl treatment, and freeze-drying	/	Glc (56.2%), Fuc (28.4%), Man (6.2%), Gal (6.0%), Xyl (1.9%), Rha (0.9%)	(1→4)-Glc, (1→3)-Fuc	→4)−Glcp−(1→	[76]

* Note: Molecular weight data for different polysaccharides are compiled from various original studies and may have been determined using different analytical methods (e.g., HPSEC-MALLS, GPC, or others). Therefore, direct quantitative comparisons of molecular weights between entries should be interpreted with caution.

**Table 2:** Molecular mechanisms of marine-derived polysaccharides against colitis. Note: This table compiles model, dosage, and mechanism data; it is not a model-stratified comparative analysis. Because DSS, TNBS, acetic acid, and cell models differ in immunological basis and translational relevance, cross-model comparisons should be interpreted cautiously.

Name	Models	Dosages	Mechanisms	References
GBP	TNBS-elicited UC Wistar rats	30, 60, 90 mg/kg	Up: GSH Down: MPO, IL-1β, TNF-α, MDA, NO_3_^−^/NO_2_^−^	[27]
BMP	DSS-stimulated IPEC-J2 cells DSS-elicited UC C57BL/6J mice	10, 20, 40 μg/mL 2 mg/kg	Up: ZO-1, Occludin, Claudin-1, IL-10 Down: FITC-D, DAO, ET-1, MPO, EPO, IL-1β, TNF-α, p-NF-κB, p-IκB-α, p-AKT	[29]
YCP	DSS-elicited UC C57BL/6 mice	40 mg/kg	Up: SOD, IL-10, IL-22, ZO-1, Claudin-1, MUC2, butyrate, isovaleric acid, Bacteroidetes, Bacteroidia, Bacteroidales Down: MDA, IL-1β, IL-6, TNF-α, Firmicutes, Proteobacteria, Clostridia, Clostridiales, Lachnospiraceae	[30]
SP	DSS-elicited UC Balb/c mice	200, 400, 600 mg/kg	Up: Claudin-1, MUC2, ZO-1, GPR43, Olfr78, GPR109A, goblet cells, Firmicutes, Actinobacteria, *Corynebacterium*, *Enterorhabdus*, *Desulfovibrio*, *Alistipes*, *Bacteroides acidifaciens* Down: ET, LBP, MPO, TNF-α, IL-1β, IL-6, acetic acid, propionic acid, butyric acid, *Bacteroides*, Acetatifactor, Defluvitaleaceae UCG-011, norank_f_Erysipelotrichaceae	[32,33]
GLP	DSS-elicited UC Kunming mice	300 mg/kg	Up: TGF-β1, CD40, goblet cells, Chao1/Shannon index, Bacteroidetes, *Lactobacillus*, *Alloprevotella*, *Prevotella*, *Ruminococcus*, total SCFAs, acetic acid, propionic acid, isovaleric acid, valeric acid Down: Simpson index, Firmicutes, Proteobacteria, Firmicutes/Bacteroidetes, CCL-25, CCR-9	[34]
PLS	Acetic acid-elicited UC Swiss mice	1, 3, 10 mg/kg	Up: GSH Down: MPO, IL-1β, TNF-α, MDA, NO_3_^−^/NO_2_^−^, iNOS	[35]
PSF-T2	LPS-stimulated RAW264.7 cells DSS-elicited UC Balb/c mice	50, 100, 200, 400 μg/mL 50, 100, 200 mg/kg	Up: MUC2, ZO-1, Occludin, E-cadherin, IκBα, goblet cells, Chao1/Shannon index, Firmicutes, Lactobacillaceae, Lachnospiraceae, Oscillospiraceae, Desulfovibrionaceae, *Lactobacillus*, *Desulfovibrio*, *Colidextribacter*, *Lachnospiraceae*_NK4A136_group, *Lachnospiraceae*_UCG-006, total SCFAs, acetic acid, propionic acid, butyric acid, isobutyric acid, valeric acid, isovaleric acid Down: IL-1β, IL-6, TNF-α, NF-κB p65, Bacteroidota, Bacteroidaceae, Erysipelotrichaceae, *Bacteroides*, *Turicibacter*	[37]
MP	LPS-stimulated RAW264.7 cells DSS-elicited UC ICR mice	100, 300, 600 μg/mL 300, 600 mg/kg	Up: GSH-Px, SOD, Chao1/Ace index, Firmicutes/Bacteroidetes, Firmicutes, Actinobacteria, *Anaerotruncus*, *Lactobacillus*, *Desulfovibrio*, *Alistipes*, *Odoribacter*, *Enterorhabdus*, *Akkermansia*, *Blauria* Down: TNF-α, IL-6, IL-10, p-p65/p65, p-IκBα/IκBα, DAO, LPS, Shannon/Simpson index, *Bacteroides*, *Romboutsia*, *Turicibacter*	[38]
JSP	DSS-elicited UC C57BL/6 mice	50, 200 mg/kg	Up: ZO-1, occludin, MUC2, E-cadherin, TFF3, goblet cells, Firmicutes/Bacteroidetes, Lactobacillaceae, Rikenellaceae, Verrucomicrobiaceae, *Akkermansia*, *Lactobacillus*, *Helicobacter*, acetate, propionate Down: TNF-α, IL-1β, IL-6, MPO, NO, FITC-D, p-p65/p65, p-IκBα/IκBα, Shannon index, Proteobacteria, Turicibacteraceae, Porphyromonadaceae, Clostridiaceae, Erysipelotrichaceae, Bradyrhizobiaceae, *Turicibacter*, *Parabacteroides*, *Clostridium*	[40]
AGSP	LPS-stimulated RAW264.7/IEC-6 cells DSS-elicited UC Balb/c mice	100 μg/mL 100, 300, 500 mg/kg	Up: IL-10, T-SOD, Lachnospiraceae, Muribaculaceae, *Muribaculum*, acetate, propionate, butyrate, isobutyrate, valerate, isovalerate Down: TNF-α, IL-1β, IL-6, MPO, NO, LPS, IKKα, IκBα, NF-κB p65, Bacteroidetes, Bacteroidaceae, Prevotellaceae, Rikenellaceae, *Bacteroides*, *Parabacteroides*, *Alistipes*	[41]
ECP	DSS-elicited UC C57BL/6J mice	100 mg/kg	Up: mucin O-glycans, *Parabacteroides* spp., *Alistipes* spp., *Parabacteroides distasonis*, *Lachnospiraceae* NK4A136 spp., *Blautia* spp., *Lactobacillus johnsonii*, Muribaculaceae, *Bacteroides ovatus*, *Bacteroides uniformis*, total SCFAs, acetate, propionate, succinate Down: lactate, *Akkermansia muciniphila*, *Bacteroides vulgatus*, *Bacteroides acidifaciens*, *Bacteroides thetaiotaomicron*, *Romboutsia ilealis*	[43,44]
REP	LPS-stimulated RAW264.7/Caco-2 cells DSS-elicited UC C57BL/6J mice	25, 50, 100 μg/mL 100 mg/kg	Up: TEER, IL-10, GSH, CAT, T-SOD, ZO-1, Occludin, Claudin1, MUC2, TFF3, E-cadherin, goblet cells, Sobs/Ace/Chao1/Shannon index, Firmicutes/Bacteroidetes, Lachnospiraceae_NK4A136_group, *Lactobacillus*, *Roseburia*, Oscillospiraceae, norank_f_Ruminococcaceae, Lachnospiraceae_UCG-010, *Bifidobacterium*, *Odoribacter*, acetate, propionate, butyrate, isobutyrate, valerate, isovalerate Down: TNF-α, IL-1β, IL-6, IL-8, IL-23, IFN-γ, FITC-D, LPS, MPO, NO, iNOS, MDA, p-p65/p65, p-IκBα/IκBα, TLR4, Simpson index, Proteobacteria, *Klebsiella*, *Escherichia-Shigella*, *Parabacteroides*, *Parasutterella*, *Veillonella*	[45,46]
OPS	DSS-elicited UC C57BL/6J mice	150, 300 mg/kg	Up: goblet cells, IL-10, ZO-1, Occludin, Claudin-4, MUC2, acetate, propionate, butyrate, HIF-1α Down: MPO, TNF-α, IL-6, LPS, TFF3	[47]
HLP	DSS-elicited UC SD rats	50, 100, 200 mg/kg	Up: IL-10, Bacteroidota, Actinobacteria, Lactobacillaceae, Oscillospiraceae, Prevotellaceae, Butyricoccaceae, *Lactobacillus*, *Alloprevotella*, *Bifidobacterium animalis*, *Corynebacterium glutamicum*, *Corynebacterium stationis*, *Faecalibaculum rodentium*, *Staphylococcus nepalensis*, butyrate, spermidine, L-tyrosine, 4-hydroxybenzoic acid, tryptophyl-tryptophan Down: TNF-α, IL-1β, IL-6, Firmicutes, Peptostreptococcaceae, Erysipelotrichaceae, Bacteroidaceae, *Bacteroides*, *Parabacteroides*, *Allobaculum*, 5-hydroxytrptamine, 5-hydroxy-3-indoleacetic acid, pyridoxal, 9,10-epoxy-18-hydroxyestearate, L-malic acid, 5-hydroxy valeric acid, Cis-9,10-epoxystearic acid	[49]
SJC	DSS-elicited UC C57BL/6J mice	0.5, 1.5, 3.5 g/kg	Up: SOD, PPAR-γ, IL-10, Occludin, ZO-1, Claudin-1, norank_f_*Muribaculaceae*, *Faecalibaculum*, *Bacteroides*, unclassified_f_*Lachnospiraceae*, *Rikenellaceae*_RC9_gut_group, *Alistipes*, total SCFAs, propionic acid, isovaleric acid Down: MDA, MPO, NF-κB, TNF-α, *Akkermansia*, Erysipelotrichaceae	[51]
SDF	DSS-elicited UC C57BL/6J mice	0.5, 1.5, 3.5 g/kg	Up: SOD, IL-10, PPAR-γ, ZO-1, Occludin, Claudin-1, Shannon/Chao1 index, Bacteroidota, Muribaculaceae, *Lachnospiraceae*_NK4A136_group, *Prevotellaceae*_UCG-001, *Coriobacteriaceae*_UCG-002, *Lactobacillus*, *Bifidobacterium*, *Faecalibaculum*, *Bacteroides*, total SCFAs, acetate, propionate, butyrate, isobutyrate, valerate, isovalerate, hexanoate Down: MDA, MPO, TNF-α, NF-κB, Firmicutes, Firmicutes/Bacteroidota, Erysipelotrichaceae, Rikenellaceae_RC9_gut_group, unclassified_f_Lachnospiraceae, *Alistipes*, *Dubosiella*, *Akkermansia*	[51]
IOP-0	DSS-elicited UC C57BL/6J mice	200, 600 mg/kg	Up: IL-10, ZO-1, Occludin, Claudin-1, Ace/Chao1/Shannon index, Bacteroidota, Campilobacterota, Proteobacteria, Muribaculaceae, Lachnospiraceae, Bifidobacteriaceae, *norank_f_Lachnospiraceae*, *Lachnospiraceae_NK4A136_group*, *Enterorhabdus*, *Escherichia-Shigella*, *Desulfovibrio*, *Rikenella*, total SCFAs, acetic acid, propionic acid, butyric acid, isobutyric acid, valeric acid, isovaleric acid, hexanoic acid, isohexanoic acid Down: TNF-α, IL-6, Simpson index, Firmicutes, Actinobacteria, Firmicutes/Bacteroidota, Erysipelotrichaceae, *Dubosella*, *Romboutsia*, *norank_f_norank_o_Clostridia_UCG-014*, *Bifidobacterium*, *Coriobacteriaceae_UCG-002*, *Saccharimonas*, *Allobaculum*, *unclassified_f_Prevotellaceae*, *Tyzzarella*	[53]
ChPS	H_2_O_2_/DSS/TNF-α/IL-1β-stimulated Caco-2 cells DSS-elicited UC C57BL/6 mice	20 μg/mL 200 mg/kg	Up: Hsp-27, Occludin, Claudin-4, ZO-1, MUC2, TFF3, goblet cells, TEER Down: ROS, MitoSOX, COX-2, iNOS, TNF-α, IL-1β, IL-6, IL-8, NF-κB, FITC-D	[54]
SpPS	H_2_O_2_/DSS/TNF-α/IL-1β-stimulated Caco-2 cells DSS-elicited UC C57BL/6 mice	20 μg/mL 200 mg/kg	Up: Hsp-27, Occludin, Claudin-4, ZO-1, MUC2, TFF3, goblet cells, TEER Down: ROS, MitoSOX, COX-2, iNOS, TNF-α, IL-1β, IL-6, IL-8, NF-κB, FITC-D	[54]
SyPS	H_2_O_2_/DSS/TNF-α/IL-1β-stimulated Caco-2 cells DSS-elicited UC C57BL/6 mice	20 μg/mL 200 mg/kg	Up: Hsp-27, Occludin, Claudin-4, ZO-1, MUC2, TFF3, goblet cells, TEER Down: ROS, MitoSOX, COX-2, iNOS, TNF-α, IL-1β, IL-6, IL-8, NF-κB, FITC-D	[54]
UPP	DSS-elicited UC BALB/c mice	50, 100, 200 mg/kg	Up: MUC2, TFF3, ZO-1, Occludin, Claudin-1, TEER, IL-4, IL-10, IgA, IκBα, goblet cells, total SCFAs, acetic acid, propionic acid, butyric acid, Bacilli, Bacteroidia, Lactobacillales, Bacteroidales, Lactobacillaceae, Muribaculaceae, *Lactobacillus* Down: MPO, iNOS, TNF-α, IL-1β, IL-6, p-ERK/ERK, p-p38/p38, Clostridia, Lachnospirares, Lachnospiraceae, *Roseburia*, *Lachnospiraceae_unclassified*, *Lachnospiraceae_NK4A136_group*	[55]
HPA	DSS-elicited UC C57BL/6J mice	200 mg/kg	Up: IL-10, ZO-1, goblet cells, Occludin, Sobs/Shannon/Ace/Chao1 index, Muribaculaceae, Atopobiaceae, Lactobacillaceae, *Bifidobacterium*, *Dubosiella*, *Clostridium UCG-014* Down: TNF-α, IL-1β, NLRP3, ASC, Caspase-1, Proteobacteria, *Escherichia-Shigella*, *Enterococcus*, *Staphylococcus*, *Clostridium_sensu_stricto_1*	[56]
EDFM	DSS-elicited UC C57BL/6 mice	19, 38, 96 mg/kg	Up: ZO-1, Occludin, MUC2, goblet cells, Ace/Chao1/Shannon/Simpson index, Firmicutes, Epsilonbacteraeota, Verrucomicrobia, *Alloprevotella*, *Akkermansia*, *Ruminiclostridium_5*, *Lachnospiraceae_NK4A136_group*, *Ruminococcaceae_UCG-014*, *uncultured_bacterium_f_Lachnospiraceae* Down: MPO, TNF-α, IL-1β, Bacteroidetes, Proteobacteria, *Escherichia-Shigella*, *Bacteroides*, *Parabacteroides*, *Enterococcus*, *Odoribacter*, *Rikenellaceae_RC9_gut_group*, *[Ruminococcus]_torques_group*	[57]
CPP	DSS-elicited UC C57BL/6J mice	25, 50, 100 mg/kg	Up: IL-10, ZO-1, Occludin, Claudin-1, MUC2, goblet cells, Bcl-2, SOD, CAT, GSH-PX, *Akkermansia*, *Bifidobacterium*, Acetovanillone, L-Tryptophan Down: MDA, MPO, COX-2, iNOS, TNF-α, IL-1β, FITC-D, DAO, Bax, Bad, Caspase-3, p-p65/p65, p-IκB/IκB, Proteobacteria, *Escherichia-Shigella*, *Parabacteroides*, *Dorea*, *Clostridium sensu stricto 1*	[58]
PTPs	DSS-elicited UC C57BL/6J mice	400, 600, 800 mg/kg	Up: IL-10, ZO-1, Occludin, Claudin-3, MUC2, goblet cells, SOD, CAT, butyrate-producing genes, Akkermansiaceae, Lactobacillaceae, *Lactobacillus*, *Muribaculum*, *Ileibacterium* Down: MDA, MPO, TNF-α, IL-1β, IL-6, IFN-γ, Claudin-2, Erysipelotrichaceae, Prevotellaceae, Oscillospiraceae, Tannerellaceae, *Prevotella*	[60]
ALP	LPS-stimulated IEC-6 cells; DSS-elicited UC Balb/c mice	25, 50, 100 μg/mL; 50, 100, 200 mg/kg	Up: Occludin, ZO-1, MUC2, Chao1/Shannon index, norank_f_Muribaculaceae, norank_o_Clostridia_UCG-014, norank_o_RF39, Candidatus_Saccharimonas, total SCFAs, butyrate, acetate, propionate Down: TNF-α, IL-1β, IL-6, LBP, *Pseudomonasota*, *Bacteroides*, *Ligilactobacillus*, unclassified_f_Enterobacteriaceae, Lachnospiraceae_NK4A136_group	[61]
UP	DSS-elicited UC C57BL/6J mice	50, 200 mg/kg	Up: *Akkermansia*, *Parabacteroides*, *Turicibacter*, f_Enterobacteriaceae, *Parabacteroides distasonis*, *Bacteroides acidifaciens*, Shannon/Simpson index, taurine, dimethylglycine Down: TNF-α, IL-1β, IL-6, *Clostridium*, *Escherichia*, Proteobacteria	[63]
Chitosan	DSS-elicited UC Kunming mice	150, 250 mg/kg	Up: ZO-1, Occludin, Claudin-1, *Blautia* sp., *Lactobacillus johnsonii*, *Lactobacillus ruminis* Down: TNF-α	[65]
Alginate	DSS-elicited UC C57BL/6 mice	5% (*w*/*w*)	Up: FXR, ACE/Chao1/Simpson index, Bifidobacterium animalis, *Lachnospiraceae NK4A136 group*, *Clostridia UCG-014*, *Faecalibaculum rodentium*, GH94, CBM50, GT5, baiJ, 7α-HSDH, 7β-HSDH, HDCA Down: TNF-α, IL-1β, IL-6, p-NF-κB p65/NF-κB p65, p-JNK1/2/JNK1/2, *Bacteroides*, *Akkermansia muciniphila*	[66]
Porphyran	DSS-elicited UC C57BL/6 mice	50 mg/kg	Up: goblet cells Down: CD11c^+^, TCR-β^+^, NK1.1^+^, Ly-6G^+^, CD11b^+^, IFN-γ^+^ CD4^+^, IL-17^+^ CD4^+^, IFN-γ^+^ CD8^+^, IL-17^+^ CD8^+^, IFN-γ, IL-17, T-bet, RORγt, CD40, CD80, CD86, IL-1β, IL-6, IL-12p70, IL-23	[67]
FVF	DSS-elicited UC C57BL/6J mice	100 mg/kg	Up: ZO-1, Occludin, MUC2, goblet cells, FXR, TGR5, Fgf15, Shp, Yap, ACE/Shannon/Sob’s index, Lachnospiraceae, Muribaculaceae, *Turicibacter*, *Muribaculum*, *Blautia*, *Parasutterella*, *Colidextribacter*, *unclassified f_Lachnospiraceae*, *GCA-900066575*, *Eubacterium xylanophilum_group*, total SCFAs, acetic acid, propionic acid, butyric acid, isovaleric acid, valeric acid, CA, TCA, GCA, UDCA, TUDCA, DCA, LCA, TLCA, TDCA, GDCA Down: MPO, TNF-α, IL-1β, IL-6, FITC-D, Lactobacillaceae, Rikenellaceae, Clostridiaceae, Peptostreptococcaceae, β-MCA, TCDCA, HDCA, GHDCA, ω-MCA, Tω-MCA, THDCA	[68]
DP-MPF	LPS-stimulated Raw264.7 cells DSS-elicited UC C57BL/6J mice	50, 100, 200 μg/mL 400 mg/kg	Up: SOD, CAT, goblet cells Down: TNF-α, IL-1α, IL-1β, IL-3, IL-6, IL-9, IL-10, IL-12(P40), IL-12(P70), IL-13, IL-17A, IFN-γ, Eotaxin, G-CSF, GM-CSF, MIP-1α, MIP-1β, MPO, NO, MDA	[69]
SLF	DSS-elicited UC BABL/c mice	100, 300 mg/kg	Up: ZO-1, Occludin, Claudin-1, CAT, T-SOD, GSH-Px, IL-10, glycoprotein, goblet cells, glycerol-3-phosphate, myo-inositol, carbamoyl phosphate, acetate, propionate, butyrate, Shannon/Simpson index, *Lachnospiraceae* NK4A136 group, *Enterorhabdus* Down: LPS, DAO, MLCK, MLC2, p-MLC2, TNF-α, IL-1β, TLR4, p65, p-p65, IκBα, p-IκBα, MPO, iNOS, MDA, N-acetyl-L-alanine, tryptophan, lactate, 2-aminooctanoic acid, AICAR, Proteobacteria, Actinobacteria, *Bacteroides*, *Romboutia*, *Turicibacter*, *Faecalibaculum*	[70]
Fuc-Sc	H_2_O_2_-stimulated Caco-2 cells DSS-elicited UC C57BL/6 mice	50, 100 μg/mL 100 mg/kg	Up: ZO-1, Claudin-1, Occludin, SOD, GSH, Nrf2, HO-1, GPX-1, NQO1, TEER Down: MDA, LDH, FITC-D	[72]
UPF	DSS-elicited UC Kunming mice	100 mg/kg	Up: IL-10, TGF-β, Shannon/Simpson index, *Bacteroidetes*, *Verrucomicrobiota*, *Lactobacillus*, *Lachnospiraceae*_NK4A136, *Alistipes*, total SCFAs, acetic acid, propionic acid, isobutyric acid, butyric acid, isovaleric acid Down: TNF-α, IL-1β, IL-6, IFN-γ, Firmicutes, Firmicutes/Bacteroidetes, *Helicobacter*, integrin α4β7, MAdCAM-1, CCR9, CCL25	[74]
Aj-FUC	DSS-elicited UC C57BL/6 mice	100, 300 mg/kg	Up: ZO-1, Occludin, MUC2, goblet cells, acetate, propionate, isobutyrate, butyrate, isovalerate, valerate, *Akkermansia*, *Lachnospiraceae*_NK4A136_group, *Lactobacillus*, *Bifidobacterium*, Bacteroidota, *Bacteroides*, *Parabacteroides* Down: TNF-α, IL-1β, IL-6, Proteobacteria, Firmicutes/Bacteroidota, Enterobacteriaceae, *Clostridia*_UCG-014, *Prevotellaceae*_UCG-001, *Escherichia-Shigella*, *Klebsiella*, *Fusobacterium*	[75]
FCC	LPS-stimulated dendritic cells DSS-elicited UC C57BL/6 mice	50 μg/mL 50 mg/kg	Up: ZO-1 Down: TCR-β^+^, CD4^+^, CD8^+^, Ly-6G^+^, TCR-γδ^+^, IFN-γ^+^ CD4, IFN-γ^+^ CD8, IL-17^+^ CD4, IFN-γ, T-bet, IL-17A, RORγt, TNF-α, IL-1β, IL-6, CD45^+^CD11c^+^, CD40, CD80, CD86, MHC I, MHC II	[76]

## Data Availability

No new data were created or analyzed in this study. Data sharing is not applicable to this article.

## Abbreviations

AKT, Protein Kinase B; ASC, apoptosis-associated speck-like protein containing a CARD; BSH, bile salt hydrolase; CAT, catalase; CCL25, C-C motif chemokine ligand 25; CCR9, C-C chemokine receptor 9; CD40, Cluster of Differentiation 40; COX-2, cyclooxygenase-2; DAI, disease activity index; DAO, diamine oxidase; DC, dendritic cell; DSS, Dextran Sulfate Sodium; EPO, eosinophil peroxidase; ERK, extracellular signal-regulated kinase; FXR, farnesoid X receptor; GPR, G protein-coupled receptor; GSH, glutathione; HDCA, hyodeoxycholic acid; HIF-1α, hypoxia-inducible factor-1 alpha; HO-1, heme oxygenase-1; 7α-HSDH, 7α-hydroxysteroid dehydrogenase; Hsp-27, Heat Shock Protein 27; IFN, Interferon; IL, interleukin; IκBα, inhibitor of nuclear factor kappa B alpha; iNOS, inducible nitric oxide synthase; JNK, c-Jun N-terminal kinase; LDH, lactate dehydrogenase; LPS, lipopolysaccharide; MAPK, mitogen-activated protein kinase; MDA, malondialdehyde; MLCK, Myosin light chain kinase; MPO, myeloperoxidase; MUC, Mucin; NF-κB, nuclear factor kappa-B; NLRP3, NOD-like receptor family pyrin domain containing 3; NO, nitric oxide; NQO1, NAD(P)H quinone dehydrogenase 1; Nrf2, nuclear factor-erythroid 2-related factor 2; PPARγ, peroxisome proliferator-activated receptor gamma; RORγt, Retinoid-related Orphan Receptor gamma t; ROS, reactive oxygen species; SCFAs, short-chain fatty acids; SOD, superoxide dismutase; T-bet, T-box transcription factor TBX21; TFF3, Trefoil Factor 3; TEER, Trans Epithelial Electrical Resistance; TGF, transforming growth factor; TGR5, takeda G protein-coupled receptor 5; Th, T helper cells; TJ, tight junction; TLR, Toll-like receptor; TNBS, 2,4,6-trinitrobenzenesulfonic acid; TNF-α, tumor necrosis factor-alpha; UC, ulcerative colitis; ZO-1, zonula occludens-1.
